# Chloride channel accessory 1 integrates chloride channel activity and mTORC1 in aging‐related kidney injury

**DOI:** 10.1111/acel.13407

**Published:** 2021-06-12

**Authors:** Hak Joo Lee, Andrew Donati, Denis Feliers, Yuyang Sun, Yanli Ding, Muniswamy Madesh, Adam B. Salmon, Yuji Ikeno, Corinna Ross, Christopher L. O'Connor, Wenjun Ju, Markus Bitzer, Yidong Chen, Goutam Ghosh Choudhury, Brij B. Singh, Kumar Sharma, Balakuntalam S. Kasinath

**Affiliations:** ^1^ Department of Medicine Center for Renal Precision Medicine University of Texas Health San Antonio TX USA; ^2^ Department of Periodontics University of Texas Health San Antonio TX USA; ^3^ Department of Pathology University of Texas Health San Antonio TX USA; ^4^ Department of Molecular Medicine University of Texas Health San Antonio TX USA; ^5^ Barshop Institute for Longevity and Aging Studies University of Texas Health San Antonio TX USA; ^6^ South Texas Veterans Health Care System San Antonio TX USA; ^7^ Geriatric Research Education & Clinical Center South Texas Veterans Health Care System San Antonio TX USA; ^8^ Texas Biomedical Research Institute Southwest National Primate Research Center San Antonio TX USA; ^9^ Department of Science and Mathematics Texas A&M University San Antonio San Antonio TX USA; ^10^ Department of Internal Medicine University of Michigan Ann Arbor MI USA; ^11^ Department of Population Health Sciences University of Texas Health San Antonio TX USA; ^12^ Greehey Children's Cancer Research Institute University of Texas Health San Antonio TX USA; ^13^ Present address: Genomic Institute of the Novartis Research Foundation La Jolla CA USA

**Keywords:** fibrosis, ion transport, senescence‐associated secretory phenotype

## Abstract

The mechanism of kidney injury in aging are not well understood. In order to identify hitherto unknown pathways of aging‐related kidney injury, we performed RNA‐Seq on kidney extracts of young and aged mice. Expression of chloride (Cl) channel accessory 1 (CLCA1) mRNA and protein was increased in the kidneys of aged mice. Immunostaining showed a marked increase in CLCLA1 expression in the proximal tubules of the kidney from aged mice. Increased kidney CLCA1 gene expression also correlated with aging in marmosets and in a human cohort. In aging mice, increased renal cortical CLCA1 content was associated with hydrogen sulfide (H_2_S) deficiency, which was ameliorated by administering sodium hydrosulfide (NaHS), a source of H_2_S. In order to study whether increased CLCA1 expression leads to injury phenotype and the mechanisms involved, stable transfection of proximal tubule epithelial cells overexpressing human CLCA1 (hCLCA1) was performed. Overexpression of hCLCA1 augmented Cl^−^ current via the Ca^++^‐dependent Cl^−^ channel TMEM16A (anoctamin‐1) by patch‐clamp studies. hCLCA1 overexpression also increased the expression of fibronectin, a matrix protein, and induced the senescence‐associated secretory phenotype (SASP). Mechanistic studies underlying these changes showed that hCLCA1 overexpression leads to inhibition of AMPK activity and stimulation of mTORC1 as cellular signaling determinants of injury. Both TMEM16A inhibitor and NaHS reversed these signaling events and prevented changes in fibronectin and SASP. We conclude that CLCA1‐TMEM16A‐Cl^−^ current pathway is a novel mediator of kidney injury in aging that is regulated by endogenous H_2_S.

## Graphical abstract

Chloride channel accessory 1 expression is increased in the tubular epithelial cells of aged kidneys. In vitro experiments show that CLCA1 augments the activity of TMEM16A, a Ca++‐dependent Chloride Channel, activates mTORC1, augments the synthesis of matrix proteins, and induces SASP, whcih contribute to aging associated kidney injury.

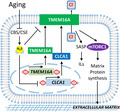

**Abbreviations**
ACC: Acetyl CoA carboxylase
AMPK: AMP‐activated protein kinase
CBS: Cystathionine 𝛃‐synthase
CLC: Chloride channel
CLCA1: Chloride channel accessory 1
CSE: Cystathionine 𝛄‐lyase
DEG: Differentially expressed genes
FN: Fibronectin
FPKM: Fragments per kilobase of transcript per million transcripts mapped
GO: Gene ontology
H2S: Hydrogen sulfide
IL: Interleukin
MCT: Murine proximal tubular epithelial cells
mTOR: Mechanistic target of rapamycin
PE: Paired‐end
SASP: Senescence associated secretory phenotype
S6K: p70 S6 kinase
TGF 𝛃1: Transforming growth factor 𝛃1
TMEM16A: Transmembrane protein 16A

## 1 INTRODUCTION

Accumulation of matrix proteins leading to glomerular and tubulointerstitial fibrosis is a major contributor to kidney injury in aging. Unabated tubulointerstitial fibrosis of the kidney is a major determinant of progressive loss of kidney function (Humphreys, 2018). These structural changes and proteinuria are evident in aging mice and aging non‐human primates (marmosets) (Bitzer & Wiggins, 2016; Lee et al., 2018, 2019; Sataranatarajan et al., 2012; Wu et al., 2010; Zheng et al., 2003). Current therapeutic approaches to inhibit kidney fibrosis are limited; while they may slow the progressive loss of kidney function, they are not able to halt it altogether. Thus, there is an urgent need to identify novel mechanisms so that interventions may be developed to arrest chronic kidney injury and functional loss encountered in aging.

Accordingly, we took a discovery approach to identify novel mRNA transcripts that may be involved in kidney aging. RNA‐Seq revealed an increase in the expression of CLCA1 mRNA in the aged kidney. CLCA1 facilitates Cl^−^ current operated by TMEM16A (anoctamin‐1), a Ca^++^‐dependent Cl^−^ channel (Jentsch & Pusch, 2018; Verkman & Galietta, 2009). Cl^−^ movement accompanied by cations such as Na^+^ and K^+^ or in exchange for other anion occurs among fluid compartments in many segments of the kidney tubule. Cl^−^ shift among these compartments is governed by electrochemical potential and transporters and exchangers.

Chloride channel accessory 1 functions as a self‐cleaving protease forming C‐terminal and N‐terminal fragments (Sala‐Rabanal et al., 2015). The N‐terminal fragment (~72 kDa) associates with the extracellular domain of TMEM16A to stimulate Cl^−^ movement in a Ca^++^‐dependent manner (Sala‐Rabanal et al., 2017). CLCA1 promotes cell membrane retention of TMEM16A rather than increase its expression (Sala‐Rabanal et al., 2015). The role played by this system has not been explored in aging‐related changes in the kidney. For the first time, we explored the role of CLCA1 in kidney injury in aging.

## 2 RESULTS

### 2.1 Chloride channel accessory 1 expression is increased in the aged kidney

**FIGURE 1:**
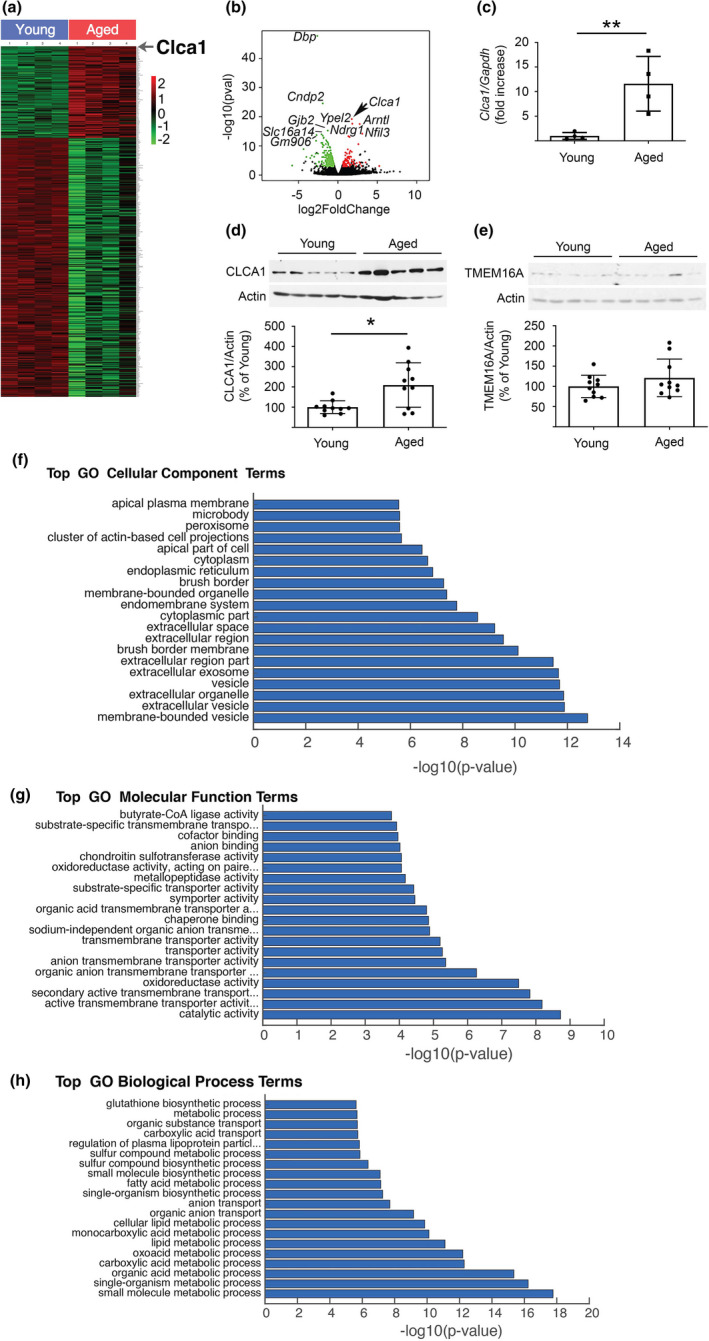
Aging is associated with an increase in kidney CLCA1 expression in mice and humans. (a) HEAT map shows the distribution of kidney mRNAs that were significantly increased (red) or decreased (green) in the aged mice (n = 4) versus young mice (n = 4). The location of CLCA1 is shown. (b) A volcano plot of mRNA changes in renal cortex of young mice and aged mice. CLCA1 expression was increased in the kidneys of aged mice (arrow). (c) RT‐qPCR using specific primers shows an increase in renal cortical CLCA1 mRNA in aged mice (n = 4) versus young mice (n = 4). (d) Immunoblotting shows an increase in CLCA1 protein expression in the renal cortex of aged mice (n = 10) compared to young mice (n = 10). (e) Immunoblotting did not show change in the expression of TMEM16A in kidneys of aged mice (n = 10) versus young mice (n = 10). (f) GO analysis of a cellular component of regulated kidney mRNAs showed the extracellular region part and extracellular space were among the major hits. (g) GO analysis for molecular function revealed anion transmembrane activity and secondary active transmembrane transporter activity among the top hits. (h) GO analysis for the biologic process identified small molecule metabolic process as one of the top findings. (c), (d), (e). Data (mean ± SD) are shown in bars with scatter plots, and were analyzed by t test. *p < 0.05, **p < 0.01

RNA‐Seq performed on the renal cortex of male young and aged mice showed that more than 400 mRNAs underwent a significant change with age (Figure 1a,b, Table S1). Top hits and their possible functional import are shown in Table S2. CLCA1 was among mRNAs that showed a robust increase with age (adjusted *p* = 4.92 × 10^−16^). We confirmed changes in CLCA1 found by RNA‐Seq by directly analyzing mRNA and protein levels in samples from aged kidneys; we confirmed that CLCA1 mRNA and the corresponding protein levels were increased in the kidney cortex of aged mice (Figure 1c,d). Aging was not associated with changes in the renal cortical expression of TMEM16A, a Ca^++^‐dependent Cl^−^ channel, the functional partner of CLCA1 (Figure 1e). Gene Ontology (GO) analysis of a cellular component of regulated renal cortical mRNAs in aged mice showed extracellular region part (Fisher's exact test, *p*‐value = 3.50 × 10^−12^) and extracellular space (Fisher's exact test, *p*‐value = 6.0 × 10^−9^) as major hits; these sites may correspond to Cl^−^ secretion that could involve CLCA1 (Figure 1f). GO analysis for molecular function revealed anion transmembrane transporter activity (Fisher's exact test, *p* = 6.40 × 10^−6^), and secondary active transmembrane transporter activity (Fisher's exact test, *p* = 1.50 × 10^−8^) among the top hits (Figure 1g); these functions also could correspond to CLCA1 as a facilitator of Cl^−^ channel activity of TMEM16A, a transmembrane protein. GO analysis of biologic process identified small molecule metabolic process as one of the top hits (Fisher's exact test, *p* = 1.70 × 10^−18^, Figure 1h), which could conceivably involve Cl^−^ transport. Thus, there was a congruence between changes in CLCA1 expression in the renal cortex of aged mice, and spatial distribution and functional processes predicted by the GO analysis.

**FIGURE 2:**
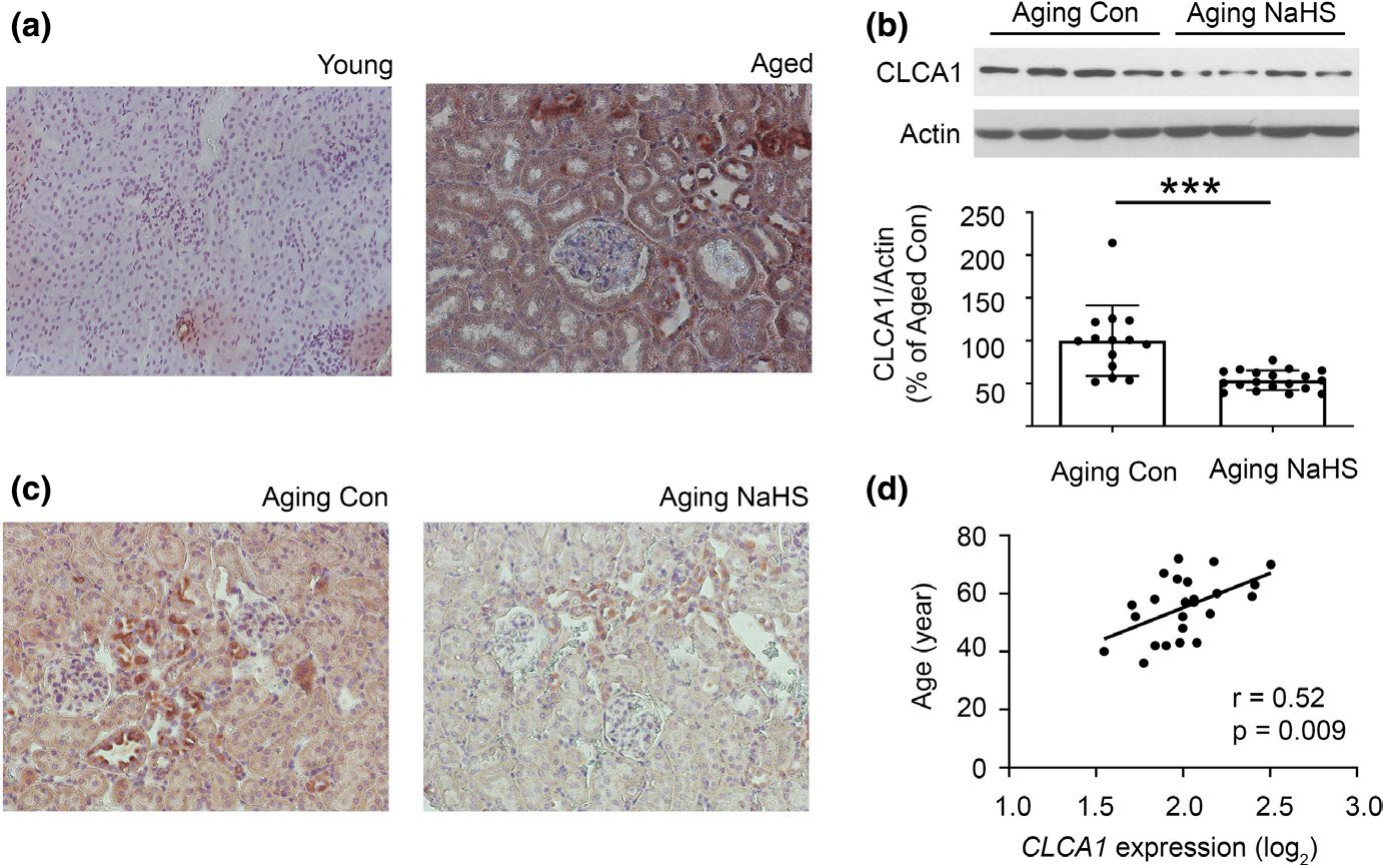
Histologic analysis of kidney CLCA1 expression. (a) On immunoperoxidase staining (x200) a faint tubular expression of CLCA1 was seen in kidney cortex from young mice, which was robustly increased in aged mice. Representative images from young (n = 4 ) and aged mice are shown (n = 4). (b, c) Administration of NaHS to 18–19‐month‐old aging mice daily for 5 months (n = 20; Aging NaHS) reduced renal cortical CLCLA1 expression by immunoblotting compared to aging mice receiving water vehicle (Aging Con; n = 14; b). This was confirmed by immunoperoxidase staining (x200; C). (d) There was a direct correlation between age and CLCA1 mRNA expression in the tubule interstitium compartment in the kidney tissue from human subjects (n = 24). (b) Data (mean ± SD) are shown in bars with scatter plots and were analyzed by t test. ***p < 0.001

Immunohistochemistry showed a faint tubular expression of CLCA1 in the renal cortex of young mice; in aged mice, CLCA1 expression was robustly increased in the tubules including in the proximal tubules; there was also some increase in the glomerular mesangium staining for CLCA1 (Figure 2a). H_2_S is constitutively synthesized in the kidney and plays a highly nuanced role in kidney physiology and pathology (Kasinath et al., 2018). In the aging kidney, deficiency in the synthesis of H_2_S is associated with accumulation of matrix proteins which contributes to renal fibrosis, inhibition of AMP‐activated protein kinase (AMPK) activity, and activation of mTORC1 (Lee et al., 2018; Sataranatarajan et al., 2012). Stimulation of mTORC1 is a major determinant of the synthesis of proteins including matrix proteins (Kasinath et al., 2009; Sataranatarajan et al., 2007). Administration of H_2_S in the form of NaHS to 18–19‐month old mice for 5 months inhibits these signaling events and an increase in matrix proteins, renal fibrosis, while also reducing albuminuria (Lee et al., 2018). These data show a fundamental role for H_2_S in aging‐associated kidney injury. We examined whether H_2_S regulated kidney CLCA1 expression in aged mice. NaHS reduced the renal cortical expression of CLCA1 in aging mice compared to untreated aging mice (Figure 2b,c).

To determine the potential evolutionary and clinical relevance of these findings, we also assessed the changes with age in CLCA1 expression in both non‐human primate and human kidney samples. Aging is associated with robust kidney fibrosis in the marmoset (*Callithrix jacchus*), a non‐human primate (Lee et al., 2019). On immunoblotting lysates from the kidney cortex of aged female marmosets, we found a significant increase in the expression of a 72 kDa fragment of CLCA1, which probably corresponds to its functional N‐terminal domain that binds to TMEM16A to augment Cl^−^ current (Figure S1); a trend toward an increase in the expression of the whole 130 kDa molecule was seen in the same animals (*p* = 0.08). The changes in kidney cortical CLCA1 expression in the aged male marmosets did not reach clinical significance. We examined if similar changes were seen in kidneys from humans. There was a significant direct correlation between age and CLCA1 mRNA expression in the kidney tubule‐interstitial compartment in a human cohort (Figure 2d); this aligned with the increased tubular expression seen in aged mice (Figure 2a). These data show the following: (1) Changes in renal cortical CLCA1 expression parallel kidney injury and its amelioration by NaHS in aging mice. (2) Tubular epithelial cells including those in the proximal tubules contribute to an increase in kidney CLCA1 expression in aging. (3) Aging‐related increase in kidney CLCA1 expression is evolutionarily conserved.

### 2.2 Chloride channel accessory 1 overexpression induces mTORC1 and augments matrix protein content

**FIGURE 3:**
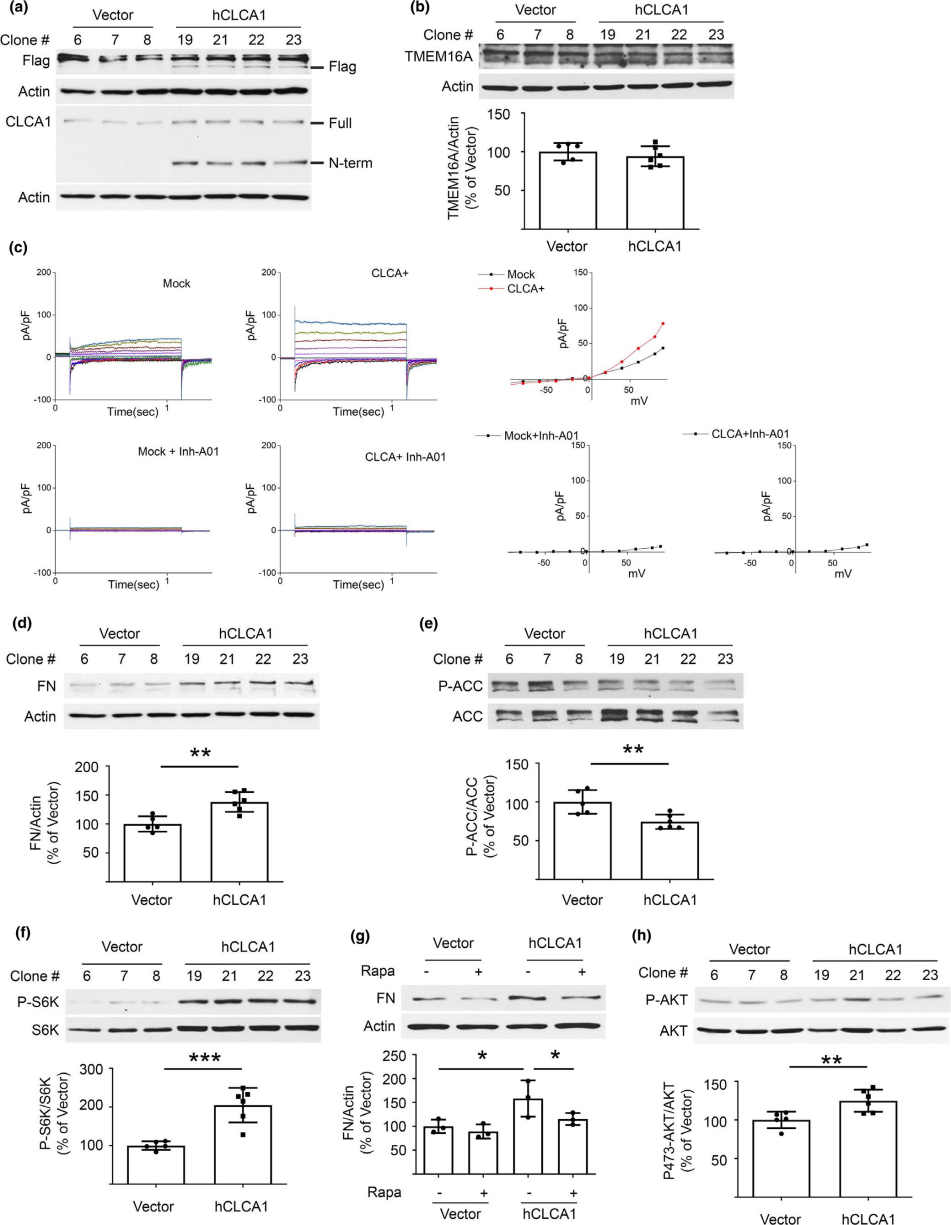
Overexpression of hCLCA1 increases matrix protein and induces changes in signaling kinases in proximal tubular epithelial cells in vitro. (a) Immunoblotting shows increased CLCA1 expression (130 kDa) and probably of its N‐terminal fragment (72 kDa) in cells stably transfected with hCLCA1 (six clones) versus control plasmid transfected clones (five clones); bands from three control and four hCLCA1 overexpressing clones are shown. (b) hCLCA1 overexpression did not alter TMEM16A expression. (c) Whole cell patch‐clamp shows an increase in Cl^−^ current in hCLCA1 overexpressing cells versus vector‐transfected controls (mock). Cl^−^ current was abolished by T16Ainh‐A01 in both mock and hCLCA1 overexpressing cells. (d) Overexpression of hCLCA1 resulted in increase in matrix protein fibronectin (FN). (e, f). Immunoblotting with specific antibodies showed decrease in ACC phosphorylation, and increase in p70S6 kinase (S6K) phosphorylation. (g) Incubation with 25 nM rapamycin for 30 min abolished the increase in fibronectin expression seen following overexpression of hCLCA1 (data from three experiments are shown). (h) Overexpression of hCLCA1 increased Akt phosphorylation at Ser473. Data (mean ± SD) are shown in bars in scatter plots and were analyzed by t test (b, d‐f, and h) or ANOVA (g). *p < 0.05, **p < 0.01, ***p < 0.001. (d, e, f, h) employed five control and six hCLCA1 clones.

We investigated if CLCA1‐TMEM16A‐Cl^−^ current system has a mechanistic role in kidney injury encountered in aging. Kidney matrix protein increase in aging mice is associated with mTORC1 activation (Lee et al., 2018). Whether CLCA1 has a role in this process is not known. We stably overexpressed FLAG‐tagged human CLCA1 (hCLCA1) in a mouse proximal tubular epithelial cell line (MCT). This resulted in increased expression of both the full length ~130 kDa CLCA1 molecule and a ~72 kDa fragment, probably the N‐terminal fragment, which associates with TMEM16A to promote Cl^−^ current (Figure 3a). However, TMEM16A expression was unchanged in hCLCA1 overexpressing cells (Figure 3b). On whole cell patch clamp, Cl^−^ current was higher in hCLCA1 overexpressing cells indicating that the Cl^−^ channel activity of TMEM16A was increased; Cl^−^ current was completely inhibited by T16Ainh‐A01, a selective TMEM16A inhibitor (Figure 3c). Overexpression of hCLCA1 resulted in increase in fibronectin, a kidney matrix protein that is increased in the aging kidney (Lee et al., 2018, 2019) (Figure 3d).

We explored signaling pathways that regulate the synthesis of proteins including matrix proteins in the kidney. AMPK is an energy sensor that constitutively inhibits mTORC1 in controlling protein synthesis in the kidney (Kasinath et al., 2009; Lee et al., 2007). hCLCA1 overexpression inhibited AMPK activity as shown by reduction in the phosphorylation of acetyl CoA carboxylase (ACC), its direct substrate (Figure 3e); increased phosphorylation of p70S6 kinase indicated mTORC1 activation (Figure 3f). Rapamycin abolished the increase in fibronectin in hCLCA1 overexpressing cells (Figure 3g) while inhibiting mTORC1 (Figure S2). Akt is upstream of mTORC1 in signaling pathways regulating protein synthesis (Kasinath et al., 2009). Akt phosphorylation of Ser473 was increased (Figure 3h) suggesting mTORC2 activation (Sarbassov et al., 2005). Akt inhibits AMPK and increases the activity of mTORC1 leading to stimulation of synthesis of proteins (Hawley et al., 2014; Kasinath et al., 2009). The reason for Akt phosphorylation increase by overexpression of CLCA1 is not clear; it is possible that it is linked to increasing Cl^−^ current at the cell surface. Changes in the phosphorylation of AMPK, mTORC1, and Akt are identical to the data reported in the renal cortex of aging mice and marmosets. (Lee et al., 2018, 2019). These results show the following: (1) Overexpression of CLCA1 increases Cl^−^ current in proximal tubular epithelial cells. (2) It also activates Akt, inhibits AMPK, and stimulates mTORC1 leading to increased fibronectin expression.

### 2.3 Chloride channel accessory 1 overexpression promotes SASP

**FIGURE 4:**
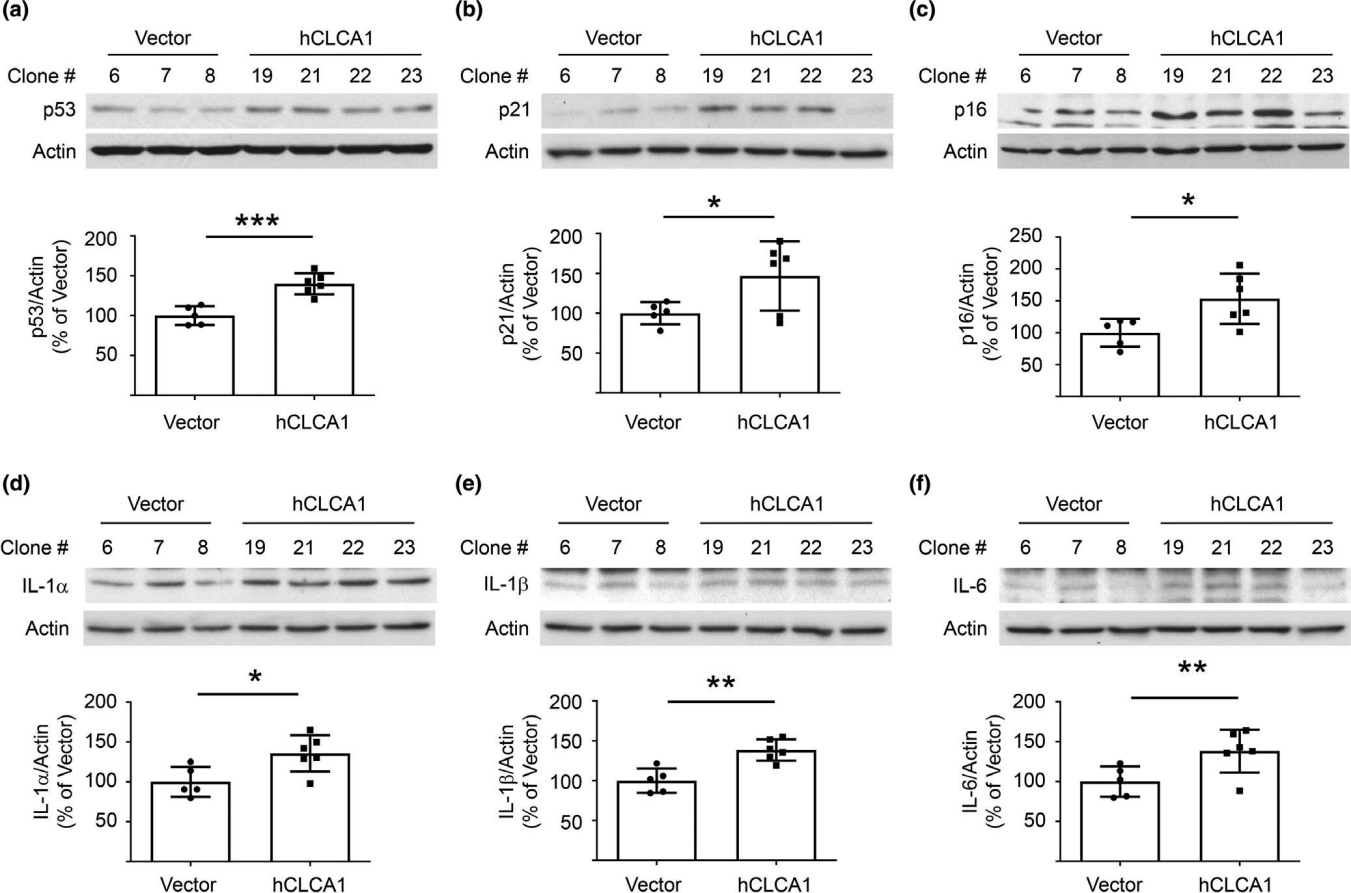
SASP is induced by overexpression of hCLCA1 in proximal tubular epithelial cells in vitro. (a‐c) Overexpression of hCLCA1 resulted in increase in the expression of p53, p21, and p16^INK4a^. (d‐f) Increased expression of IL‐1α, IL‐1β, and IL‐6 was seen in cells overexpressing hCLCA1. Data (mean ± SD) are shown in bars with scatter plots and were analyzed by t test. *p < 0.05, **p < 0.01, ***p < 0.001. (a‐f, five control and six hCLCA1 clones were employed)

A subset of kidney cells including proximal tubular epithelial cells manifest SASP in aging mice, and interventions that reduce SASP ameliorate aging‐associated kidney injury (Baker et al., 2016; Lee et al., 2018). SASP has been linked to mTOR activation (Herranz et al., 2015). SASP involves the secretion of pro‐fibrotic factors that promote kidney fibrosis (Xu et al., 2020). Wnt9–TGFβ1 pathway has been shown to be involved in this process (Luo et al., 2018). hCLCA1 overexpressing cells showed increased expression of p53, p21, and p16^INK4a^, features of cell senescence (Figure 4a‐c). Cells manifesting SASP assume a pro‐inflammatory phenotype; kidney content of IL‐1β and IL‐6 is increased in aging mice (Lee et al., 2018) Overexpression of hCLCA1 augmented the expression of IL‐1α, IL‐1β, and IL‐6 (Figure 4d‐f) in agreement with the in vivo observations. Data in Figures 3 and 4 show that hCLCA1 overexpression reproduces key aging‐associated changes in kidney matrix proteins, signaling pathways, and SASP. These data implicate CLCA1 system in bringing about these aging‐associated features in the kidney.

### 2.4 TMEM16A activity is required for CLCA1‐induced changes

**FIGURE 5:**
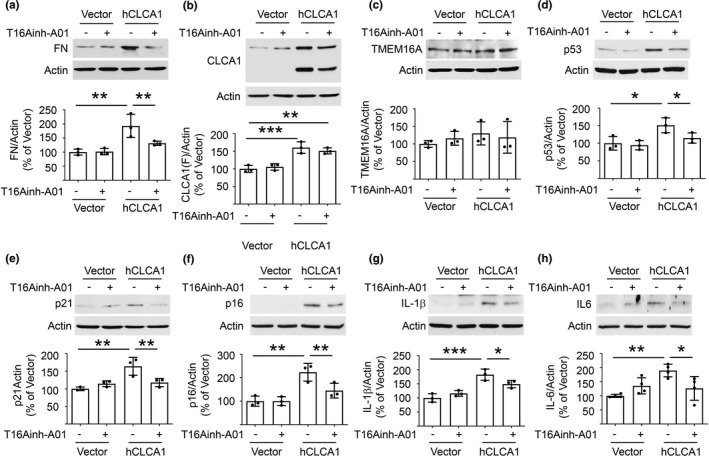
TMEM16A inhibition abolishes changes in fibronectin and SASP induced by overexpression of hCLCA1 in proximal tubular epithelial cells in vitro. (a‐c) T16Ainh‐A01, a selective TMEM16A inhibitor, inhibited increase in matrix fibronectin induced by hCLCA1 overexpression versus vector‐transfected controls without affecting the expression of CLCA1 and TMEM16A. (d‐h) T16Ainh‐A01 abolished hCLCA1‐overexpression‐induced increase in p53, p21, p16^INK4a^, IL‐1β, and IL‐6. Data from 3–4 experiments (mean ± SD) are shown in scatter plots and were analyzed by ANOVA. *p < 0.05, **p < 0.01, ***p < 0.001

Chloride channel accessory 1 acts to facilitate Cl^−^ current generated by TMEM16A. Alternatively, it could have TMEM16A‐independent actions. We explored whether the effects of CLCA1 overexpression required the activity of TMEM16A by employing T16Ainh‐A01, a TMEM16A selective inhibitor that blocks the activity of the latter (Figure 3c). The inhibitor abolished the increase in fibronectin induced by hCLCA1 overexpression without affecting the expression of hCLCA1 or TMEM16A (Figure 5a‐c). T16Ainh‐A01 also inhibited features of SASP induced by hCLCA1 overexpression (Figure 5d‐h). These data demonstrate that the ability of CLCA1 to stimulate matrix protein synthesis and induce SASP in renal epithelial cells requires the activation of TMEM16A, the Ca^++^‐dependent Cl^−^ channel.

### 2.5 Effect of H_2_S on CLCA1‐induced changes

**FIGURE 6:**
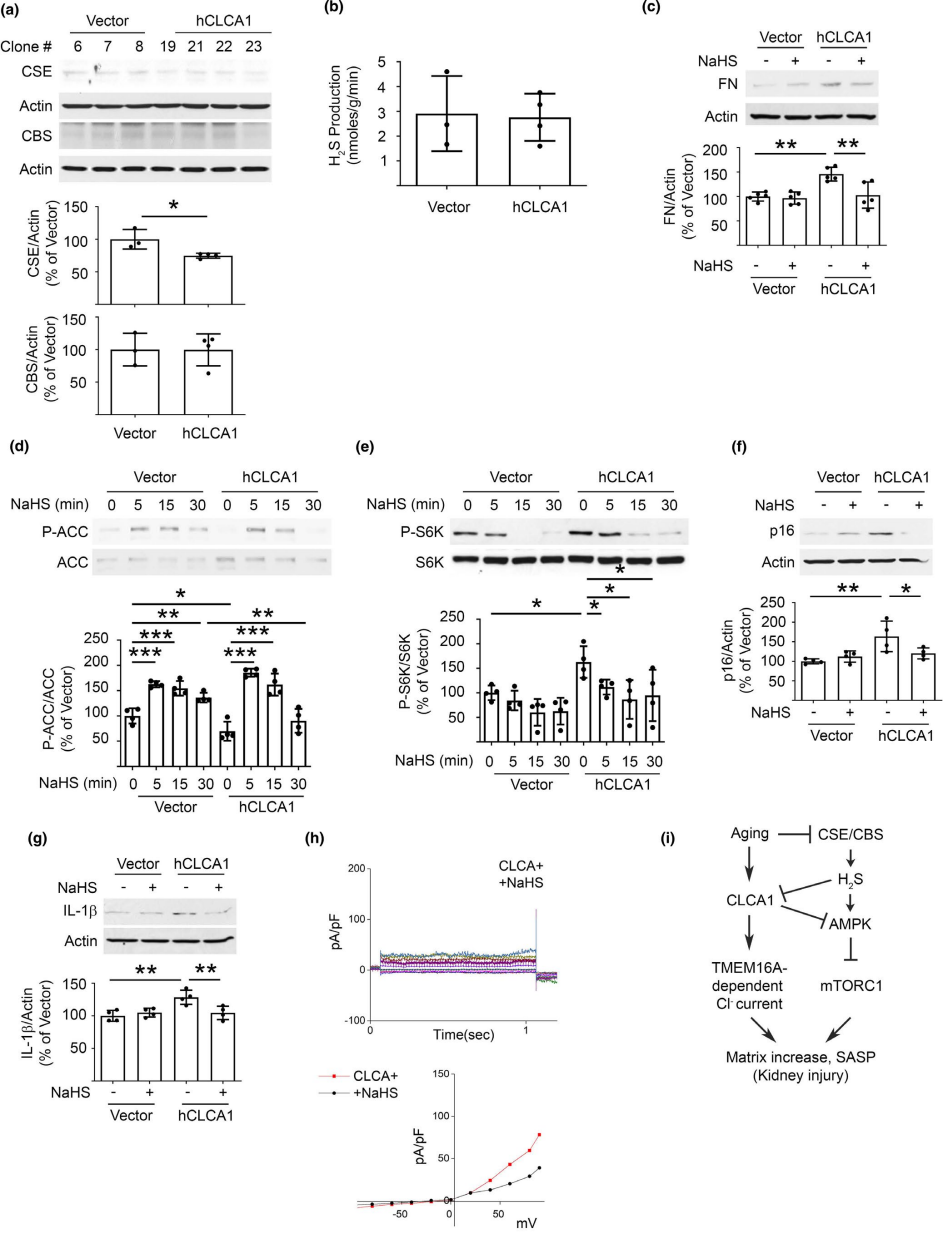
Interaction between overexpression of hCLCA1 and H_2_S in proximal tubular epithelial cells in vitro. (a) Expression of CSE but not CBS, enzymes in the trans‐sulfuration pathway involved in H_2_S generation, was decreased by overexpression of hCLCA1. (b) Increased hCLCA1 expression did not affect H_2_S generation in MCT cells. (c) NaHS abolished the increase in fibronectin expression induced by overexpression of hCLCA1. (d) AMPK activity as assessed by phosphorylation of its substrate ACC was inhibited by overexpression of hCLCA1, which was abolished by NaHS. (e) Activity of mTORC1 as evaluated by phosphorylation of its substrate p70S6 kinase(S6K) was increased by hCLCA1 overexpression, which was reduced by NaHS. (f, g) NaHS inhibited some aspects of SASP, that is, increase in p16^INK4a^ and IL‐1β, induced by overexpression of hCLCA1. Data from four experiments (mean ± SD) are shown in bars with scatter plots and were analyzed by ANOVA. *p < 0.05, **p < 0.01, ***p < 0.001. (h) Patch‐clamp studies: addition of NaHS inhibited Cl^−^ current induced by hCLCA‐1 overexpression. (i) A schematic summarizes the role of CLCA1‐TMEM16A‐Cl^−^ current in aging‐associated kidney injury.

Recent findings indicate that gaseous molecules including H_2_S are important regulators of kidney function (Feliers et al., 2016; Kasinath et al., 2018). H_2_S is constitutively synthesized by the kidney (Lee et al., 2018, 2019). Proximal tubule epithelial cells and podocytes synthesize H_2_S in vitro (Lee et al., ,^2012^, ^2017^). Cystathionine γ‐lyase (CSE) and cystathionine β‐synthase (CBS) are involved in H_2_S generation in the trans‐sulfuration pathway (Kasinath et al., 2018). Kidney injury manifesting as matrix accumulation in diabetes is associated with the decreased synthesis of H_2_S (Lee et al., 2012); administration of H_2_S ameliorates kidney injury in diabetic rodents (Zhou et al., 2014). We have reported that kidney H_2_S generation is decreased in aging mice, and that administration of NaHS ameliorates matrix protein increase and SASP in them (Lee et al., 2018). We tested if there was an interaction between CLCA1 and H_2_S. Overexpression of hCLCA1 reduced the expression of CSE but not of CBS; however, H_2_S generation was unaffected (Figure 6a,b) suggesting that CLCA1‐independent mechanisms cause H_2_S deficiency in the aging kidney. Interestingly, NaHS inhibited an increase in fibronectin induced by hCLCA1 overexpression (Figure 6c). We examined the signaling mechanism underlying NaHS effects. NaHS countered changes in the activity of AMPK and mTORC1 seen with overexpression of hCLCA1. Thus, NaHS restored AMPK activity and inhibited mTORC1 activity in hCLCA1 overexpressing cells (Figure 6d,e). Additionally, NaHS abolished some aspects of SASP in hCLCA1 overexpressing cells (Figure 6f,g). These effects of H_2_S are in agreement with the data in aging mice that received NaHS (Lee et al., 2018). Overexpression of hCLCA1 resulted in an increase in Cl^−^ current (Figure 3c). Patch‐clamp studies showed that hCLCA1‐induced Cl^−^ current was inhibited by NaHS (Figure 6h). Together, in vivo and in vitro data identify CLCA1‐TMEM16A‐Cl^−^ current axis as an important regulator of matrix accumulation and SASP in aging. H_2_S is upstream of CLCA1 as supported by the abolition of increase in kidney CLCA1 expression by the administration of NaHS to aging mice (Figure 2b,c), and, by inhibition of Cl^−^ current induced by CLCA1 overexpression (Figure 6h).

## 3 DISCUSSION

For the first time, we show that CLCA1 contributes to kidney injury in aging by stimulating Cl^−^ current via activation of TMEM16A, a Ca^++^‐dependent Cl^−^ channel. Activation of Cl^−^ current, a cell surface phenomenon, sets off signaling events that regulate the synthesis of proteins and induce SASP in proximal tubular epithelial cells (Figure 6i). Our additional novel finding is that this system is under the regulation of H_2_S.

Different types of Cl^−^ channels (CLC) exist in the kidney (Verkman & Galietta, 2009). Eight members of CLC gene family serve as anion channels or anion‐proton exchangers (Jentsch & Pusch, 2018). Other types of Cl^−^ channels in the kidney include cystic fibrosis transmembrane conductance regulator, Ca^++^‐dependent Cl^−^ channels, for example, TMEM16A (anoctamin‐1), volume‐regulated anion channels, ligand‐gated channels, and maxi‐ion channels. They are involved in endosomal and lysosomal functions, and Golgi acidification (Jentsch & Pusch, 2018; Verkman & Galietta, 2009). Our study involves the TMEM16A channel.

TMEM16A belongs to a family of ten members (TMEM16A‐K) (Caputo et al., 2008; Schroeder et al., 2008; Yang et al., 2013). TMEM16A is expressed in airway epithelia and smooth muscle, and is linked to mucus cell metaplasia and airway hyperactivity (Caputo et al., 2008; Huang et al., 2012; Scudieri et al., 2012). In the kidney, podocytes, proximal tubular epithelial cells, and principal epithelial cells of collecting ducts express TMEM16A (Faria et al., 2014; Schreiber et al., 2019). It is located in the apical and sub‐apical membrane domains in proximal tubular epithelial cells (Faria et al., 2014). In addition to being a Cl^−^ channel, TMEM16A regulates albumin uptake and endosomal acidification (Faria et al., 2014). Global TMEM16A knock‐out mice develop albuminuria and die postnatally due to unstable airway (Ousingsawat et al., 2009; Rock et al., 2008). Podocyte‐specific TMEM16A knock‐out mice display normal podocyte structure and glomerular filtration associated with non‐significant increase in albuminuria (Faria et al., 2014). Tubule‐specific TMEM16A knock‐out mice develops transient proteinuria associated with retention of endosomal vesicles in proximal tubules (Faria et al., 2014). Combined podocyte and tubular TMEM16A knock‐out mice have reduced glomerular number and proteinuria; proximal tubular epithelial cells show loss of cilia (Schenk et al., 2018). TMEM16A is involved in polycystic kidney disease by increasing Cl^−^ secretion and cyst growth (Buchholz et al., 2014; Schreiber et al., 2019). ATP, UTP, calmodulin, phosphatidylinositol 4,5‐bisphosphate, ezrin, radixin, and moesin regulate TMEM16A activity (Perez‐Cornejo et al., 2012; Pritchard et al., 2014; Schwiebert et al., 2002; Tian et al., 2011; Turner et al., 2007). Activation of TMEM16A is facilitated by CLCA1 which has four human and eight murine homologs; CLCA1 is secreted (Gibson et al., 2005; Mundhenk et al., 2018; Yurtsever et al., 2012). Cells in colon, small intestine, and kidney express CLCA1 (Agnel et al., 1999).

We report for the first time that kidney cortical CLCA1 expression is increased in aging as an evolutionarily conserved feature. Patch‐clamp studies in proximal tubular epithelial cells in vitro showed that overexpression of hCLCA1 resulted in increase in Cl^−^ current by TMEM16A activation. Increased hCLCA1 expression markedly affected signaling leading to increased matrix protein content. hCLCA1 overexpression drove Akt phosphorylation which was associated with downstream stimulation of mTORC1, and an increase in matrix proteins. Similar processes of activation of Akt‐mTORC1 axis also occur in association with fibrosis in the kidney in aging mice (Lee et al., 2018). Inhibition of TMEM16A mitigated mTORC1 activation and increase in matrix proteins in hCLCA1 overexpressing cells. These data show CLCA1‐TMEM16A‐Cl^−^ axis is upstream of signaling regulation of matrix protein synthesis in the aged kidney. SASP induction by overexpression of CLCA1 may involve mTORC1 activation, a known inducer of SASP (Herranz et al., 2015). A key issue for future investigations is how the process of cell surface anion secretion should trigger SASP.

Interestingly, we found an interaction between the gasotransmitter H_2_S and the CLCA1‐TMEM16A‐Cl^−^ current system. H_2_S is drawing increasing attention for its ability to regulate several critical biological functions of the kidney (Kasinath et al., 2018). As mentioned in the results section, the kidney normally generates significant amounts of H_2_S. Reduced H_2_S generation occurs in several models of kidney injury including acute kidney injury due to ischemia, urinary tract obstruction, or *cis*‐platinum toxicity, and, also in chronic kidney injury encountered in diabetes and hypertension including pregnancy‐related preeclampsia. Treatment with H_2_S donors ameliorates kidney injury in these disorders (Kasinath et al., 2018). Our previous work has added kidney injury due to aging to this list. Aging kidneys have decreased expression of CSE and CBS enzymes leading to reduced H_2_S generation. Administration of H_2_S to aging mice ameliorates fibrosis, albuminuria, and SASP by stimulating AMPK and inhibiting mTORC1 in the kidney (Lee et al., 2018). Our studies on mechanisms underlying the salutary effect of H_2_S on cell injury caused by CLCA1 overexpression also revealed that it involves stimulation of AMPK and inhibition of mTORC1. These data together demonstrate that H_2_S deficiency is a proximal event in the aging kidney leading to downstream activation of CLCA1‐TMEM16A‐Cl^−^ current axis and mTORC1‐regulated cell events. The latter include matrix protein synthesis and SASP (Figure 6i) as both are linked to mTOR activation (Herranz et al., 2015; Kasinath et al., 2009). SASP has been linked to extracellular matrix remodeling and kidney fibrosis by activation of the pro‐fibrotic pathways including the Wnt9 and TGFβ1 pathways (Luo et al., 2018). We saw similar recruitment of AMPK by H_2_S to inhibit mTOR and matrix protein increase in high glucose‐treated podocytes (Lee et al., 2012). In addition, high glucose‐induced increase in NADPH oxidase 4 expression and subsequent oxidative injury in proximal tubule epithelial cells was also inhibited by H_2_S by activating AMPK (Lee et al., 2017). These data suggest that H_2_S employs a common signaling mechanism involving AMPK activation in protecting against kidney cell injury.

The mechanism by which stimulation of CLCA1‐TMEM16A pathway activates Akt signaling is not clear from our studies. Increased Cl^−^ current may be associated with Na^+^ and fluid secretion across the cell membrane altering cell shape, which can activate signaling pathways (Schmick & Bastiaens, 2014). Studies are needed to address the status of Cl^−^ secretion in the aging kidney; data on measurement of Cl^−^ in excreted urine will be difficult to interpret because of numerous other factors that regulate Cl^−^ transport in and out of tubular cells in the kidney. These non‐CLCA1‐TMEM16A determinants of Cl^−^ handling in the kidney include many other types of Cl^−^ channels, Na^+^ K^+^ 2Cl transporter, Cl^−^ anion exchangers to mention a few. Additionally, passive paracellular Cl^−^ transport mechanisms also exist in the kidney. Urinary Cl^−^ is a composite result of all these determinants.

TMEM16A promotes cyst growth in polycystic kidney disease (Schreiber et al., 2019). Aging is associated with the development of kidney cysts; CLCA1‐TMEM16A‐Cl^−^ current axis could have a role in this phenomenon. Kidney cysts are common in end‐stage kidney disease, often associated with malignancy. These processes could also involve the CLCA1‐TMEM16A‐Cl^−^ current system.

In summary, we identify CLCA1‐TMEM16A‐Cl^−^ current axis as a novel paradigm contributing to kidney injury in aging. It could serve as an interventional target to arrest aging‐associated kidney injury.

## 4 EXPERIMENTAL PROCEDURES

### 4.1 RNA‐Seq for Transcriptome Profiling and Bioinformatics

RNA was extracted from the kidney cortex of C57BL6 young mice (n = 4, mean age 5 months) and aged mice (n = 4, mean age 32 months), using TRIzol (Cat. #, 15596018, Thermo Fisher Scientific) as previously described (Cavaglieri et al., 2015). RNA quality and integrity were checked using Agilent 2100 Bioanalyzer using RNA 6000 Nano kit (Agilent Technologies). Approximately 500 ng total RNA was used for RNA‐Seq library preparation by following the KAPA stranded RNA‐Seq kit with RiboErase (HMR) sample preparation guide (Cat. #, KR1151, KAPA Biosystems). The first step in the workflow involved the depletion of rRNA by hybridization of complementary DNA oligonucleotides, followed by treatment with RNase H and DNase to remove rRNA duplexed to DNA and original DNA oligonucleotides, respectively. Following rRNA removal, the remaining RNA was fragmented into small pieces using divalent cations under elevated temperature and magnesium. The cleaved RNA fragments were copied into first strand cDNA using reverse transcriptase and random primers. This was followed by second strand cDNA synthesis using DNA polymerase I and RNase H. Strand specificity was achieved by replacing dTTP with dUTP in the second strand marking mix . The incorporation of dUTP in the second strand synthesis effectively quenches the second strand during amplification, since the polymerase used in the assay will not incorporate past this nucleotide. These cDNA fragments then went through an end repair process, the addition of a single ‘A’ base, and then ligation of the adapters. The products were then purified and enriched with PCR to create the final RNA‐Seq library. RNA‐Seq libraries were subjected to quantification process by the combination of Qubit and Bioanalyzer, pooled for cBot amplification and subsequent sequenced with 100 bp paired‐end (PE) sequencing module with Illumina HiSeq 3000 platform. After the sequencing run, demultiplexing with script bcl2fastq2 (Illumina) were employed to generate the fastq file for each sample. The average of 41M PE reads was generated for each sample. All sequencing reads were aligned with their reference genome (UCSC mouse genome build mm9) using TopHat2 (Trapnell et al., 2012) with options (‐r 100 ‐‐no‐sort‐bam–no‐coverage‐search ‐‐library‐type fr‐firststrand) and the BAM files obtained after alignment were processed using HTSeq‐count (Anders et al., 2015) (with options: ‐s reverse ‐q ‐i gene_id) using NCBI RefSeq gene annotation for mm9 to obtain the counts per gene in all samples. Expression abundance of each gene was subsequently converted in unit of the fragment per kilobase of transcript per million transcripts mapped (FPKM). Differential expression was evaluated using DESeq (Anders & Huber, 2010) to obtain fold‐change, *p*‐value, and adjusted *p*‐value using Benjamini and Hochberg, (1995) correction for multiple tests. We selected differentially expressed genes (DEGs) based on the following criteria: 1) fold‐change >1.5) adjusted *p*‐value <0.05; and 3) FPKM >1. Gene ontology enrichment from the DEG list was performed using TopGO package (Alexa et al., 2006), and significantly enriched functions were selected based on classic Fisher's Exact test *p*‐value <0.05. Differential expression was visualized, such as the volcano plot, using R (https://www.r‐project.org). Data are available at https://www.ncbi.nlm.nih.gov/geo/query/acc.cgi?acc=GSE155407.

### 4.2 mRNA measurement by real‐time (RT)‐qPCR

We employed the same RNA with RNA‐Seq analysis for RT‐qPCR. Total RNA was treated with DNase I for 30 min at 37°C, and then the mixture was incubated for 10 min at 80℃ to inactivate DNase I. 1 µg of RNA was used for cDNA generation using iScript RT SuperMix for RT‐qPCR (Cat. #, 1708841, Bio‐Rad). 2 µl of cDNAs were used for RT‐qPCR using RT^2^ SYBR Green ROX^™^ RT‐qPCR Mastermix (Cat. #, 330520, Qiagen) and primers for mouse CLCA1 (Cat. # PPK040451E‐200, Qiagen), mouse *Tmem16A* (Cat. # PPM26917B‐200, Qiagen) and mouse glyceraldehyde‐3 phosphate dehydrogenase (*Gapdh*; Cat. # PPM02946E, Qiagen). The RT‐qPCR was run in a MasterCycler RealPlex4 (Eppendorf). Quantitation of the mRNAs was performed using the 2^−ΔΔCt^ method using *Gapdh* as a housekeeping gene (Cavaglieri et al., 2015).

### 4.3 Cell Culture

Murine proximal tubular epithelial (MCT) cells (kindly provided by Dr. Eric Neilson, Northwestern University, Chicago, IL) were grown in Dulbecco's modified Eagle's medium containing 7% fetal bovine serum, 5.5 mM glucose, 100 units/ml penicillin, 100 µg/ml streptomycin, and 2 mM glutamine (Mariappan et al., 2005).

### 4.4 Generation of human chloride channel accessory 1 expressing cells

Transfection was performed employing FuGENE^®^HD according to the manufacturer's instructions (Promega Corporation). One µg of pCMV6‐hCLCA1 plasmid (Cat. #, RC218583, OriGene Technologies Inc) or pCMV‐entry plasmid (Cat. #, PS100001, OriGene Technologies Inc) were diluted in Opti‐MEM (Cat. #, 31985070, Thermo Fisher Scientific). 1 × 10^5^ of MCT cells were seeded onto a 12‐well plate and transfected using 6 µl of FuGENE^®^HD transfection reagent (Cat. #, E2312, Promega Corporation) for 24 h. The cells were transferred into 6 × 96‐well plates and incubated with 800 µg/ml of G418 (Cat. #, 4727878001, MiliporeSigma) for 7 days to select single clones. Single clones were expanded onto 100 mm plates for 2 weeks with 800 µg/ml of G418. hCLCA1 expression was determined in 12 vector clones and 32 h CLCA1 clones using antibodies against CLCA1 and FLAG (DDK). Five vector clones and six hCLCA1 clones were chosen for experiments.

### 4.5 Animals

Animal experiments employing mice and marmosets were approved by the Institutional Animal Care and Use Committee of the University of Texas Health, San Antonio, TX, and, the Southwest National Primate Research Center at the Texas Biomedical Research Institute, San Antonio, TX, respectively.

### 4.6 Aging mice

We employed 5‐month old (n = 10) and 30‐month old (n = 10) male C57BL6 mice (inhouse breeding) as young and aged, respectively. We also used kidney preparations from a previously reported study in which 18‐ and 19‐month‐old male C57BL6 mice (National Institute of Aging) were randomized to receive 30 µmol/L of NaHS (Cat. #, 161527, Millipore Sigma) in drinking water (n = 20) or plain water (n = 14) for 5 months as previously described (Lee et al., 2018).

### 4.7 Aged marmosets

We have recently reported aging‐associated changes in the kidneys of marmoset, a non‐human primate (Lee et al., 2019). To study changes in kidney CLCA1 expression in aged marmosets, we performed immunoblotting on kidney cortical lysates from four male young (average age 3 years), four female young (average age 3 years), five aged male (average age 16 years), and five aged female marmosets (average age 16 years).

### 4.8 Human kidneys

Kidney tissue was obtained from the unaffected parts of kidneys removed from patients undergoing surgery at the University of Michigan and processed via the tissue procurement service of the Department of Pathology. Clinical data were obtained through the honest broker office of the University of Michigan. Tissue was placed right away in RNAlater, micro‐dissected into glomeruli and tubule‐interstitial fractions, and isolated RNA was used for gene expression analysis using Affymetrix Human Gene 2.1 ST Array (Hodgin et al., 2015; Kato et al., 2016). This study was approved by the Institutional Review Board of the University of Michigan.

### 4.9 Immunohistochemistry

Mouse kidneys were fixed in 10% formaldehyde and embedded in paraffin, and 4‐μm sections were cut. Sections were incubated with Xylene‐S (Cat. # HC‐700, Thermo Fisher Scientific) to remove paraffin and washed by ethanol. After deparaffinization, the slides were incubated with antigen unmasking solution (Cat. #, H3300, Vector Laboratories) at 125°C for 30 s and 90°C for 10 s using a decloacking chamber (Cat. #, DC2002, Biocare Medical LLC). The staining was performed by Vectastain ABC kit protocol (Cat. #, PK‐4001, Vector Laboratories). CLCA1 antibodies were diluted (1:50) in Davinci Green diluent (Cat. #, PD‐900L, Biocare Medical). After incubation with Vectastain ABC kit, the slides were incubated with ImmPACT NovaRED peroxidase substrate (Cat. #, SK‐4805, Vector Laboratories) for 3–4 min at RT followed by hematoxylin staining for 2 min at RT.

### 4.10 Immunoblotting

Equal amount of protein lysates or renal cortical homogenates were analyzed by immunoblotting (Lee et al., 2010, 2015). We employed antibodies against the following; CLCA1 (Cat. #, ab180851, Abcam Plc), TMEM16A (Cat. #, ab72984, Abcam Plc), flag (Cat. #, MA1‐142, Thermo Fisher Scientific), fibronectin (Cat. #, ab2413, Abcam Plc), collagen 1α2 (Cat. #, 14695–1‐AP, Proteintech Group Inc), phospho‐Ser79‐acetyl‐CoA carboxylase (ACC; Cat. #, 3661, Cell Signaling Technology), ACC (Cat. #, 3662, Cell Signaling Technology), phospho‐Ser473‐Akt (Cat. #, 9271, Cell Signaling Technology), Akt (Cat. #, 9272, Cell Signaling Technology), phospho‐Thr389‐p70S6 kinase (Cat. #, 9205, Cell Signaling Technology), p70S6 kinase (Cat. #, 9202, Cell Signaling Technology), p53 (Cat. #, sc‐6243, Santa Cruz Biotechnology), p21 (Cat. #, ab109199, Abcam Plc), p16^INK4a^ (Cat. #, ab189034, Abcam Plc), IL‐1α (Cat. #, ab7632, Abcam Plc), IL‐1β (Cat. #, ab9722, Abcam Plc), IL‐6 (Cat. #, ab7737, Abcam Plc), and Actin (Cat. #, A2066, Millipore Sigma).

### 4.11 Cl^−^ current measurement

Patch‐clamp experiments were done as previously described (Sun et al., 2015). Coverslips with cells were transferred to the recording chamber, and perfused with an external Ringer's solution of the following composition: Tetraethylammonium‐Cl, 145 mM; MgCl_2_, 1 mM; CaCl_2_, 2 mM; Hepes, 10 mM; Glucose, 10 mM; pH 7.3 was adjusted with Tris. Whole cell currents were recorded using an Axopatch 200B (Axon Instruments, Inc.). The patch pipette had resistances between 3 and 5 MΩ after filling with the standard intracellular solution that contained the following: TEA‐Cl, 145 mM; MgATP, 3 mM; Hepes, 10 mM; EGTA, 5 mM; pH 7.2 was adjusted with Tris. Total CaCl_2_ was adjusted to 300 nM. Free Ca^2+^ was calculated using WebmaxC Standard (http://www.stanford.edu/∼cpatton/webmaxcS.htm). The stimulation protocol to generate current–voltage relationships consisted in 2 s‐long voltage steps from −100 to +100 mV in 20 mV increments starting from a holding potential of −60 mV. Currents were recorded at 2 kHz and digitized at 5–8 kHz. pClamp version 10.1 software was used for data acquisition and analysis. The basal leak was subtracted from the final currents and average currents are shown. All experiments were carried out at room temperature.

### 4.12 Statistics

Data were expressed as mean ± SD; analyses between two groups were performed by the t test using the GraphPad Prism. Data were considered statistically significant at *p* < 0.05. Statistical comparisons between multiple subgroups were performed by ANOVA one‐way, and post hoc analysis was done using Tukey multiple comparisons test, employing GraphPad Prism version 8.

## CONFLICTS OF INTEREST

Kumar Sharma is on the Data Safety Monitoring Board of Sanofi and Cara Therapeutics. All other authors declare that they have no conflict of interest.

## AUTHOR CONTRIBUTIONS

BSK supervised the project in its conception, design, data interpretation, and wrote the manuscript. HJL contributed to the design and conducted experiments with the aid of AD. YS and BBS designed and conducted patch‐clamp experiments. AS, YI, CR, YD, CLO, WJ, and MB helped in collecting and interpreting data on kidney samples from experimental animals and humans. YC supervised RNA‐Seq data collection and interpretation. DF, GGC, MM, BBS, YC, and KS participated in data interpretation, and writing of the manuscript. All authors provided input and approved the manuscript.

### Open Research Badges

This article has been awarded Open Materials, and Open Data Badges. All materials and data are publicly accessible via the Open Science Framework at https://www.ncbi.nlm.nih.gov/geo/query/acc.cgi?acc=GSE155407.

## Supporting information

Supplementary Material

Supplementary Material

## Data Availability

RNA‐Seq data are available at: https://www.ncbi.nlm.nih.gov/geo/query/acc.cgi?acc=GSE155407. Cultured proximal tubule cells stably transfected with hCLCA1 are available on request.

## References

[acel13407-bib-0001] Agnel, M. , Vermat, T. , & Culouscou, J. M. (1999). Identification of three novel members of the calcium‐dependent chloride channel (CaCC) family predominantly expressed in the digestive tract and trachea. FEBS Letters, 455, 295–301. 10.1016/s0014-5793(99)00891-1 10437792

[acel13407-bib-0002] Alexa, A. , Rahnenfuhrer, J. , & Lengauer, T. (2006). Improved scoring of functional groups from gene expression data by decorrelating GO graph structure. Bioinformatics, 22, 1600–1607. 10.1093/bioinformatics/btl140 16606683

[acel13407-bib-0003] Anders, S. , & Huber, W. (2010). Differential expression analysis for sequence count data. Genome Biology, 11, R106. 10.1186/gb-2010-11-10-r106 20979621 PMC3218662

[acel13407-bib-0004] Anders, S. , Pyl, P. T. , & Huber, W. (2015). HTSeq–a python framework to work with high‐throughput sequencing data. Bioinformatics, 31, 166–169. 10.1093/bioinformatics/btu638 25260700 PMC4287950

[acel13407-bib-0005] Baker, D. J. , Childs, B. G. , Durik, M. , Wijers, M. E. , Sieben, C. J. , Zhong, J. , A. Saltness, R. , Jeganathan, K. B. , Verzosa, G. C. , Pezeshki, A. , Khazaie, K. , Miller, J. D. , & van Deursen, J. M. (2016). Naturally occurring p16(Ink4a)‐positive cells shorten healthy lifespan. Nature, 530, 184–189. 10.1038/nature16932 26840489 PMC4845101

[acel13407-bib-0006] Benjamini, Y. , & Hochberg, Y. (1995). Controlling the false discovery rate: A practical and powerful approach to multiple testing. Journal of the Royal Statistical Society: Series B (Methodological), 57, 289–300. 10.1111/j.2517-6161.1995.tb02031.x

[acel13407-bib-0007] Bitzer, M. , & Wiggins, J. (2016). Aging biology in the kidney. Advances in Chronic Kidney Disease, 23, 12–18. 10.1053/j.ackd.2015.11.005 26709058

[acel13407-bib-0008] Buchholz, B. , Schley, G. , Faria, D. , Kroening, S. , Willam, C. , Schreiber, R. , Klanke, B. , Burzlaff, N. , Jantsch, J. , Kunzelmann, K. , & Eckardt, K. U. (2014). Hypoxia‐inducible factor‐1alpha causes renal cyst expansion through calcium‐activated chloride secretion. Journal of the American Society of Nephrology, 25, 465–474. 10.1681/ASN.2013030209 24203996 PMC3935579

[acel13407-bib-0009] Caputo, A. , Caci, E. , Ferrera, L. , Pedemonte, N. , Barsanti, C. , Sondo, E. , Pfeffer, U. , Ravazzolo, R. , Zegarra‐Moran, O. , & Galietta, L. J. V. (2008). TMEM16A, a membrane protein associated with calcium‐dependent chloride channel activity. Science, 322, 590–594. 10.1126/science.1163518 18772398

[acel13407-bib-0010] Cavaglieri, R. C. , Day, R. T. , Feliers, D. , & Abboud, H. E. (2015). Metformin prevents renal interstitial fibrosis in mice with unilateral ureteral obstruction. Molecular and Cellular Endocrinology, 412, 116–122. 10.1016/j.mce.2015.06.006 26067231

[acel13407-bib-0011] Faria, D. , Rock, J. R. , Romao, A. M. , Schweda, F. , Bandulik, S. , Witzgall, R. , Schlatter, E. , Heitzmann, D. , Pavenstädt, H. , Herrmann, E. , Kunzelmann, K. , & Schreiber, R. (2014). The calcium‐activated chloride channel Anoctamin 1 contributes to the regulation of renal function. Kidney International, 85, 1369–1381. 10.1038/ki.2013.535 24476694

[acel13407-bib-0012] Feliers, D. , Lee, H. J. , & Kasinath, B. S. (2016). Hydrogen sulfide in renal physiology and disease. Antioxidants & Redox Signaling, 25, 720–731. 10.1089/ars.2015.6596 27005700 PMC5079410

[acel13407-bib-0013] Gibson, A. , Lewis, A. P. , Affleck, K. , Aitken, A. J. , Meldrum, E. , & Thompson, N. (2005). hCLCA1 and mCLCA3 are secreted non‐integral membrane proteins and therefore are not ion channels. Journal of Biological Chemistry, 280, 27205–27212. 10.1074/jbc.M504654200 15919655

[acel13407-bib-0014] Hawley, S. A. , Ross, F. A. , Gowans, G. J. , Tibarewal, P. , Leslie, N. R. , & Hardie, D. G. (2014). Phosphorylation by Akt within the ST loop of AMPK‐alpha1 down‐regulates its activation in tumour cells. The Biochemical Journal, 459, 275–287. 10.1042/BJ20131344 24467442 PMC4052680

[acel13407-bib-0015] Herranz, N. , Gallage, S. , Mellone, M. , Wuestefeld, T. , Klotz, S. , Hanley, C. J. , Raguz, S. , Acosta, J. C. , Innes, A. J. , Banito, A. , Georgilis, A. , Montoya, A. , Wolter, K. , Dharmalingam, G. , Faull, P. , Carroll, T. , Martínez‐Barbera, J. P. , Cutillas, P. , Reisinger, F. , … Gil, J. (2015). mTOR regulates MAPKAPK2 translation to control the senescence‐associated secretory phenotype. Nature Cell Biology, 17, 1205–1217. 10.1038/ncb3225 26280535 PMC4589897

[acel13407-bib-0016] Hodgin, J. B. , Bitzer, M. , Wickman, L. , Afshinnia, F. , Wang, S. Q. , O'Connor, C. , Yang, Y. , Meadowbrooke, C. , Chowdhury, M. , Kikuchi, M. , Wiggins, J. E. , & Wiggins, R. C. (2015). Glomerular aging and focal global glomerulosclerosis: A podometric perspective. Journal of the American Society of Nephrology: JASN, 26, 3162–3178. 10.1681/ASN.2014080752 26038526 PMC4657829

[acel13407-bib-0017] Huang, F. , Zhang, H. , Wu, M. , Yang, H. , Kudo, M. , Peters, C. J. , Woodruff, P. G. , Solberg, O. D. , Donne, M. L. , Huang, X. , Sheppard, D. , Fahy, J. V. , Wolters, P. J. , Hogan, B. L. M. , Finkbeiner, W. E. , Li, M. , Jan, Y.‐N. , Jan, L. Y. , & Rock, J. R. (2012). Calcium‐activated chloride channel TMEM16A modulates mucin secretion and airway smooth muscle contraction. Proceedings of the National Academy of Sciences of the USA, 109, 16354–16359. 10.1073/pnas.1214596109 22988107 PMC3479591

[acel13407-bib-0018] Humphreys, B. D. (2018). Mechanisms of renal fibrosis. Annual Review of Physiology, 80, 309–326. 10.1146/annurev-physiol-022516-034227 29068765

[acel13407-bib-0019] Jentsch, T. J. , & Pusch, M. (2018). CLC Chloride channels and transporters: Structure, function, physiology, and disease. Physiological Reviews, 98, 1493–1590. 10.1152/physrev.00047.2017 29845874

[acel13407-bib-0020] Kasinath, B. S. , Feliers, D. , & Lee, H. J. (2018). Hydrogen sulfide as a regulatory factor in kidney health and disease. Biochemical Pharmacology, 149, 29–41. 10.1016/j.bcp.2017.12.005 29225129

[acel13407-bib-0021] Kasinath, B. S. , Feliers, D. , Sataranatarajan, K. , Ghosh Choudhury, G. , Lee, M. J. , & Mariappan, M. M. (2009). Regulation of mRNA translation in renal physiology and disease. American Journal of Physiology. Renal Physiology, 297, F1153–F1165. 10.1152/ajprenal.90748.2008 19535566 PMC2781325

[acel13407-bib-0022] Kato, M. , Wang, M. , Chen, Z. , Bhatt, K. , Oh, H. J. , Lanting, L. , Deshpande, S. , Jia, Y. E. , Lai, J. Y. C. , O’Connor, C. L. , Wu, Y. F. , Hodgin, J. B. , Nelson, R. G. , Bitzer, M. , & Natarajan, R. (2016). An endoplasmic reticulum stress‐regulated lncRNA hosting a microRNA megacluster induces early features of diabetic nephropathy. Nature Communications, 7, 12864. 10.1038/ncomms12864 PMC5553130 27686049

[acel13407-bib-0023] Lee, H. J. , Feliers, D. , Barnes, J. L. , Oh, S. , Choudhury, G. G. , Diaz, V. , Galvan, V. , Strong, R. , Nelson, J. , Salmon, A. , Kevil, C. G. , & Kasinath, B. S. (2018). Hydrogen sulfide ameliorates aging‐associated changes in the kidney. Geroscience, 40, 163–176. 10.1007/s11357-018-0018-y 29717417 PMC5964063

[acel13407-bib-0024] Lee, H. J. , Feliers, D. , Mariappan, M. M. , Sataranatarajan, K. , Choudhury, G. G. , Gorin, Y. , & Kasinath, B. S. (2015). Tadalafil integrates nitric oxide‐hydrogen sulfide signaling to inhibit high glucose‐induced matrix protein synthesis in podocytes. Journal of Biological Chemistry, 290, 12014–12026. 10.1074/jbc.M114.615377 PMC4424338 25752605

[acel13407-bib-0025] Lee, H. J. , Gonzalez, O. , Dick, E. J. , Donati, A. , Feliers, D. , Choudhury, G. G. , Ross, C. , Venkatachalam, M. , Tardif, S. D. , & Kasinath, B. S. (2019). Marmoset as a model to study kidney changes associated with aging. Journals of Gerontology. Series A, Biological Sciences and Medical Sciences, 74, 315–324. 10.1093/gerona/gly237 PMC6376089 30321310

[acel13407-bib-0026] Lee, H. J. , Lee, D. Y. , Mariappan, M. M. , Feliers, D. , Ghosh‐Choudhury, G. , Abboud, H. E. , Gorin, Y. , & Kasinath, B. S. (2017). Hydrogen sulfide inhibits high glucose‐induced NADPH oxidase 4 expression and matrix increase by recruiting inducible nitric oxide synthase in kidney proximal tubular epithelial cells. The Journal of Biological Chemistry, 292, 5665–5675. 10.1074/jbc.M116.766758 28188286 PMC5392562

[acel13407-bib-0027] Lee, H. J. , Mariappan, M. M. , Feliers, D. , Cavaglieri, R. C. , Sataranatarajan, K. , Abboud, H. E. , Choudhury, G. G. , & Kasinath, B. S. (2012). Hydrogen sulfide inhibits high glucose‐induced matrix protein synthesis by activating AMP‐activated protein kinase in renal epithelial cells. Journal of Biological Chemistry, 287, 4451–4461. 10.1074/jbc.M111.278325 PMC3281646 22158625

[acel13407-bib-0028] Lee, M.‐J. , Feliers, D. , Mariappan, M. M. , Sataranatarajan, K. , Mahimainathan, L. , Musi, N. , Foretz, M. , Viollet, B. , Weinberg, J. M. , Choudhury, G. G. , & Kasinath, B. S. (2007). A role for AMP‐activated protein kinase in diabetes‐induced renal hypertrophy. American Journal of Physiology Renal Physiology, 292, F617–F627. 10.1152/ajprenal.00278.2006 17018841

[acel13407-bib-0029] Lee, M.‐J. , Feliers, D. , Sataranatarajan, K. , Mariappan, M. M. , Li, M. , Barnes, J. L. , Choudhury, G. G. , & Kasinath, B. S. (2010). Resveratrol ameliorates high glucose‐induced protein synthesis in glomerular epithelial cells. Cellular Signalling, 22, 65–70. 10.1016/j.cellsig.2009.09.011 19765649 PMC2766430

[acel13407-bib-0030] Luo, C. , Zhou, S. , Zhou, Z. , Liu, Y. , Yang, L. I. , Liu, J. , Zhang, Y. , Li, H. , Liu, Y. , Hou, F. F. , & Zhou, L. (2018). Wnt9a promotes renal fibrosis by accelerating cellular senescence in tubular epithelial cells. Journal of the American Society of Nephrology, 29, 1238–1256. 10.1681/ASN.2017050574 29440280 PMC5875944

[acel13407-bib-0031] Mariappan, M. M. , Senthil, D. , Natarajan, K. S. , Choudhury, G. G. , & Kasinath, B. S. (2005). Phospholipase Cgamma‐Erk Axis in vascular endothelial growth factor‐induced eukaryotic initiation factor 4E phosphorylation and protein synthesis in renal epithelial cells. Journal of Biological Chemistry, 280, 28402–28411. 10.1074/jbc.M504861200 15919658

[acel13407-bib-0032] Mundhenk, L. , Erickson, N. A. , Klymiuk, N. , & Gruber, A. D. (2018). Interspecies diversity of chloride channel regulators, calcium‐activated 3 genes. PLoS One, 13, e0191512. 10.1371/journal.pone.0191512 29346439 PMC5773202

[acel13407-bib-0033] Ousingsawat, J. , Martins, J. R. , Schreiber, R. , Rock, J. R. , Harfe, B. D. , & Kunzelmann, K. (2009). Loss of TMEM16A causes a defect in epithelial Ca2+‐dependent chloride transport. Journal of Biological Chemistry, 284, 28698–28703. 10.1074/jbc.M109.012120 PMC2781414 19679661

[acel13407-bib-0034] Perez‐Cornejo, P. , Gokhale, A. , Duran, C. , Cui, Y. , Xiao, Q. , Hartzell, H. C. , & Faundez, V. (2012). Anoctamin 1 (Tmem16A) Ca2+‐activated chloride channel stoichiometrically interacts with an ezrin‐radixin‐moesin network. Proceedings of the National Academy of Sciences USA, 109, 10376–10381. 10.1073/pnas.1200174109 PMC3387097 22685202

[acel13407-bib-0035] Pritchard, H. A. , Leblanc, N. , Albert, A. P. , & Greenwood, I. A. (2014). Inhibitory role of phosphatidylinositol 4,5‐bisphosphate on TMEM16A‐encoded calcium‐activated chloride channels in rat pulmonary artery. British Journal of Pharmacology, 171, 4311–4321. 10.1111/bph.12778 24834965 PMC4241096

[acel13407-bib-0036] Rock, J. R. , Futtner, C. R. , & Harfe, B. D. (2008). The transmembrane protein TMEM16A is required for normal development of the murine trachea. Developmental Biology, 321, 141–149. 10.1016/j.ydbio.2008.06.009 18585372

[acel13407-bib-0037] Sala‐Rabanal, M. , Yurtsever, Z. , Berry, K. N. , Nichols, C. G. , & Brett, T. J. (2017). Modulation of TMEM16A channel activity by the von Willebrand factor type A (VWA) domain of the calcium‐activated chloride channel regulator 1 (CLCA1). Journal of Biological Chemistry, 292, 9164–9174. 10.1074/jbc.M117.788232 PMC5454099 28420732

[acel13407-bib-0038] Sala‐Rabanal, M. , Yurtsever, Z. , Nichols, C. G. , & Brett, T. J. (2015). Secreted CLCA1 modulates TMEM16A to activate Ca(2+)‐dependent chloride currents in human cells. Elife, 4, e05875. 10.7554/eLife.05875 PMC4360653 25781344

[acel13407-bib-0039] Sarbassov, D. D. , Guertin, D. A. , Ali, S. M. , & Sabatini, D. M. (2005). Phosphorylation and regulation of Akt/PKB by the rictor‐mTOR complex. Science, 307, 1098–1101. 10.1126/science.1106148 15718470

[acel13407-bib-0040] Sataranatarajan, K. , Feliers, D. , Mariappan, M. M. , Lee, H. J. , Lee, M. J. , Day, R. T. , Yalamanchili, H. B. , Choudhury, G. G. , Barnes, J. L. , Van Remmen, H. , Richardson, A. , & Kasinath, B. S. (2012). Molecular events in matrix protein metabolism in the aging kidney. Aging Cell, 11, 1065–1073. 10.1111/acel.12008 23020145 PMC5812369

[acel13407-bib-0041] Sataranatarajan, K. , Mariappan, M. M. , Lee, M. J. , Feliers, D. , Choudhury, G. G. , Barnes, J. L. , & Kasinath, B. S. (2007). Regulation of elongation phase of mRNA translation in diabetic nephropathy: Amelioration by rapamycin. American Journal of Pathology, 171, 1733–1742. 10.2353/ajpath.2007.070412 PMC2111098 17991718

[acel13407-bib-0042] Schenk, L. K. , Buchholz, B. , Henke, S. F. , Michgehl, U. , Daniel, C. , Amann, K. , Kunzelmann, K. , & Pavenstädt, H. (2018). Nephron‐specific knockout of TMEM16A leads to reduced number of glomeruli and albuminuria. American Journal of Physiology Renal Physiology, 315, F1777–F1786. 10.1152/ajprenal.00638.2017 30156115

[acel13407-bib-0043] Schmick, M. , & Bastiaens, P. I. H. (2014). The interdependence of membrane shape and cellular signal processing. Cell, 156, 1132–1138. 10.1016/j.cell.2014.02.007 24630717

[acel13407-bib-0044] Schreiber, R. , Buchholz, B. , Kraus, A. , Schley, G. , Scholz, J. , Ousingsawat, J. , & Kunzelmann, K. (2019). Lipid peroxidation drives renal cyst growth in vitro through activation of TMEM16A. Journal of the American Society of Nephrology, 30, 228–242. 10.1681/ASN.2018010039 30606785 PMC6362630

[acel13407-bib-0045] Schroeder, B. C. , Cheng, T. , Jan, Y. N. , & Jan, L. Y. (2008). Expression cloning of TMEM16A as a calcium‐activated chloride channel subunit. Cell, 134, 1019–1029. 10.1016/j.cell.2008.09.003 18805094 PMC2651354

[acel13407-bib-0046] Schwiebert, E. M. , Wallace, D. P. , Braunstein, G. M. , King, S. R. , Peti‐Peterdi, J. , Hanaoka, K. , Guggino, W. B. , Guay‐Woodford, L. M. , Bell, P. D. , Sullivan, L. P. , Grantham, J. J. , & Taylor, A. L. (2002). Autocrine extracellular purinergic signaling in epithelial cells derived from polycystic kidneys. American Journal of Physiology Renal Physiology, 282, F763–F775. 10.1152/ajprenal.0337.2000 11880338

[acel13407-bib-0047] Scudieri, P. , Caci, E. , Bruno, S. , Ferrera, L. , Schiavon, M. , Sondo, E. , Tomati, V. , Gianotti, A. , Zegarra‐Moran, O. , Pedemonte, N. , Rea, F. , Ravazzolo, R. , & Galietta, L. J. V. (2012). Association of TMEM16A chloride channel overexpression with airway goblet cell metaplasia. Journal of Physiology, 590, 6141–6155. 10.1113/jphysiol.2012.240838 PMC3530122 22988141

[acel13407-bib-0048] Sun, Y. , Birnbaumer, L. , & Singh, B. B. (2015). TRPC1 regulates calcium‐activated chloride channels in salivary gland cells. Journal of Cellular Physiology, 230, 2848–2856. 10.1002/jcp.25017 25899321 PMC4872598

[acel13407-bib-0049] Tian, Y. , Kongsuphol, P. , Hug, M. , Ousingsawat, J. , Witzgall, R. , Schreiber, R. , & Kunzelmann, K. (2011). Calmodulin‐dependent activation of the epithelial calcium‐dependent chloride channel TMEM16A. The FASEB Journal, 25, 1058–1068. 10.1096/fj.10-166884 21115851

[acel13407-bib-0050] Trapnell, C. , Roberts, A. , Goff, L. , Pertea, G. , Kim, D. , Kelley, D. R. , Pimentel, H. , Salzberg, S. L. , Rinn, J. L. , & Pachter, L. (2012). Differential gene and transcript expression analysis of RNA‐seq experiments with tophat and cufflinks. Nature Protocols, 7, 562–578. 10.1038/nprot.2012.016 22383036 PMC3334321

[acel13407-bib-0051] Turner, C. M. , King, B. F. , Srai, K. S. , & Unwin, R. J. (2007). Antagonism of endogenous putative P2Y receptors reduces the growth of MDCK‐derived cysts cultured in vitro. American Journal of Physiology Renal Physiology, 292, F15–25. 10.1152/ajprenal.00103.2006 16849696

[acel13407-bib-0052] Verkman, A. S. , & Galietta, L. J. (2009). Chloride channels as drug targets. Nature Reviews Drug Discovery, 8, 153–171. 10.1038/nrd2780 19153558 PMC3601949

[acel13407-bib-0053] Wu, J. , Zhang, R. , Torreggiani, M. , Ting, A. , Xiong, H. , Striker, G. E. , Vlassara, H. , & Zheng, F. (2010). Induction of diabetes in aged C57B6 mice results in severe nephropathy: An association with oxidative stress, endoplasmic reticulum stress, and inflammation. American Journal of Pathology, 176, 2163–2176. 10.2353/ajpath.2010.090386 PMC2861082 20363923

[acel13407-bib-0054] Xu, J. , Zhou, L. , & Liu, Y. (2020). Cellular senescence in kidney fibrosis: Pathologic significance and therapeutic strategies. Frontiers in Pharmacology, 11, 601325. 10.3389/fphar.2020.601325 33362554 PMC7759549

[acel13407-bib-0055] Yang, B. , Cao, L. , Liu, B. , McCaig, C. D. , & Pu, J. (2013). The transition from proliferation to differentiation in colorectal cancer is regulated by the calcium activated chloride channel A1. PLoS One, 8, e60861. 10.1371/journal.pone.0060861 23593331 PMC3625186

[acel13407-bib-0056] Yurtsever, Z. , Sala‐Rabanal, M. , Randolph, D. T. , Scheaffer, S. M. , Roswit, W. T. , Alevy, Y. G. , Patel, A. C. , Heier, R. F. , Romero, A. G. , Nichols, C. G. , Holtzman, M. J. , & Brett, T. J. (2012). Self‐cleavage of human CLCA1 protein by a novel internal metalloprotease domain controls calcium‐activated chloride channel activation. Journal of Biological Chemistry, 287, 42138–42149. 10.1074/jbc.M112.410282 PMC3516759 23112050

[acel13407-bib-0057] Zheng, F. , Plati, A. R. , Potier, M. , Schulman, Y. , Berho, M. , Banerjee, A. , Leclercq, B. , Zisman, A. , Striker, L. J. , & Striker, G. E. (2003). Resistance to glomerulosclerosis in B6 mice disappears after menopause. American Journal of Pathology, 162, 1339–1348. 10.1016/S0002-9440(10)63929-6 PMC1851217 12651625

[acel13407-bib-0058] Zhou, X. , Feng, Y. , Zhan, Z. , & Chen, J. (2014). Hydrogen sulfide alleviates diabetic nephropathy in a streptozotocin‐induced diabetic rat model. The Journal of Biological Chemistry, 289, 28827–28834. 10.1074/jbc.M114.596593 25164822 PMC4200243

