# Supplementary material for: Chloride channel accessory 1 integrates chloride channel activity and mTORC1 in aging‐related kidney injury
Source: Aging Cell. 2021 Jun 12;20(7):e13407. doi: 10.1111/acel.13407 (PMC8282273; doi:10.1111/acel.13407)
Supplement: Supplementary file 1 — Supplementary Material [file ACEL-20-e13407-s001.pdf]

Supplemental Material Table of Contents

Supplementary Figures

S1. Aging is associated with increased kidney CLCA1 expression in marmosets. Immunoblotting of kidney cortex of young and aged female marmosets showed increased expression of the 72 kDa fragment of CLCA1, probably the N-terminal fragment. A trend towards increase in the expression of the whole 130 kDa molecule was seen in the same animals.

S2. Rapamycin abolished the increase in phosphorylation of p70S6 kinase in hCLCA1 overexpressing cells indicating inhibition of mTORC1.

Supplementary Tables

Table 1. List of mRNAs that were differentially regulated in renal cortex of aged mice.

Table 2. Top hits on RNA Seq and their possible functions.

Supplementary Figure S1

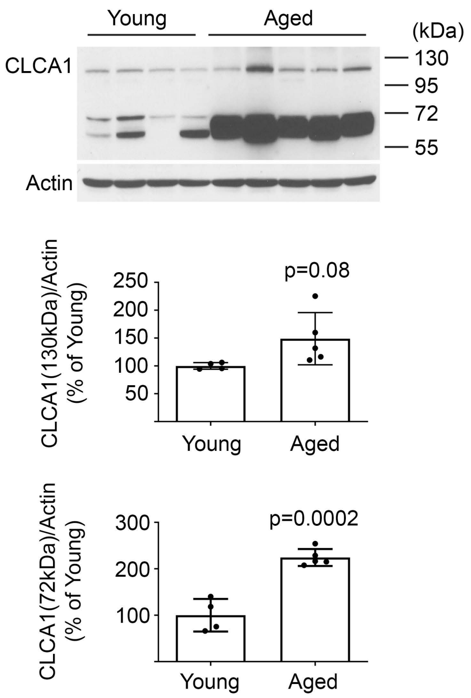

Supplementary Figure S2

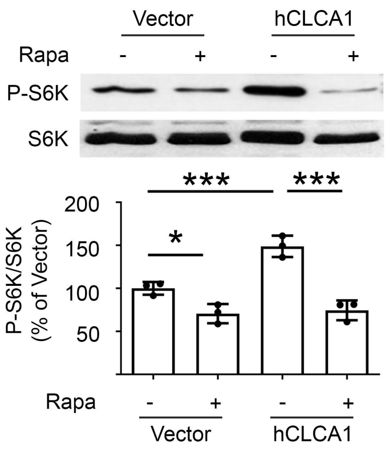

Supplementary Table 1

List of mRNAs that were differentially regulated in renal cortex of aged mice

id	baseMean	baseMean1_Group_Y	baseMean2_Group_O	FC_2over1	log2FC_2over1	pval	padj	geneName
Dbp	1053.722954	1818.008972	289.4369365	0.15920545	-2.651038371	2.61E-48	4.76E-44	D site albumin promoter binding protein
Cndp2	18768.89938	29859.92537	7677.87339	0.257129691	-1.959431884	3.72E-25	3.39E-21	CNDP dipeptidase 2 (metallopeptidase M20 family)
Clca1	418.327617	195.4522148	641.2030193	3.280612705	1.713965286	8.10E-20	4.92E-16	chloride channel accessory 1
Ypel2	682.6427859	307.7383851	1057.547187	3.436513733	1.780945725	3.19E-18	1.46E-14	yippee-like 2 (Drosophila)
Arntl	334.2584058	88.12686545	580.3899461	6.585845793	2.719368732	4.16E-18	1.52E-14	aryl hydrocarbon receptor nuclear translocator-like
Gjb2	2238.481135	3202.099596	1274.862675	0.398133361	-1.328676329	7.32E-16	2.22E-12	gap junction protein, beta 2
Nfil3	193.7958882	41.84139841	345.750378	8.263356177	3.046727854	8.66E-15	2.25E-11	nuclear factor, interleukin 3, regulated
Slc16a14	1951.855307	3170.863062	732.8475515	0.231119268	-2.113290552	1.39E-14	3.17E-11	solute carrier family 16 (monocarboxylic acid transporters), member 14
Gm906	48.01323846	83.57120313	12.45527379	0.149037866	-2.746249171	2.55E-14	5.17E-11	predicted gene 906
Tmem169	189.7720272	301.7255606	77.81849373	0.257911506	-1.955051958	4.28E-14	7.81E-11	transmembrane protein 169
Ndrg1	55689.10339	32387.72184	78990.48493	2.438902165	1.286231887	6.44E-14	1.07E-10	N-myc downstream regulated gene 1
Akr1c13	356.9682268	193.1849289	520.7515246	2.695611544	1.43061261	1.14E-13	1.73E-10	aldo-keto reductase family 1, member C13
Nudt19	11940.37592	18677.27912	5203.472722	0.278599077	-1.843737617	1.83E-13	2.56E-10	nudix (nucleoside diphosphate linked moiety X)-type motif 19
Acy3	21915.42855	34395.79716	9435.05993	0.274308512	-1.866128705	2.79E-13	3.63E-10	aspartoacylase (aminoacylase) 3
Prr15	200.5091269	297.7277547	103.2904991	0.346929359	-1.52728616	1.57E-12	1.91E-09	proline rich 15
Acsm3	8521.756246	14920.21303	2123.299466	0.142310265	-2.812888368	2.35E-12	2.68E-09	acyl-CoA synthetase medium-chain family member 3
Csad	2942.984882	4288.492332	1597.477432	0.372503273	-1.424674992	4.80E-12	5.14E-09	cysteine sulfinic acid decarboxylase
Coq10b	746.9477279	1069.591696	424.3037597	0.396696946	-1.333890803	7.86E-12	7.96E-09	coenzyme Q10B
Gm853	678.456766	1105.99482	250.9187125	0.226871508	-2.140052658	1.22E-11	1.13E-08	predicted gene 853
Dio1	223.2363075	357.9162532	88.55636174	0.247422018	-2.014954202	1.24E-11	1.13E-08	deiodinase, iodothyronine, type I
Gna14	318.7571489	450.9807839	186.5335139	0.413617432	-1.273631104	2.17E-11	1.88E-08	guanine nucleotide binding protein, alpha 14
Col15a1	561.942779	839.4688946	284.4166633	0.338805482	-1.561470877	3.07E-11	2.54E-08	collagen, type XV, alpha 1
Fgb	41.38870721	12.05472344	70.72269098	5.866803278	2.552574617	3.37E-11	2.67E-08	fibrinogen beta chain
Chst7	341.2781169	544.4383707	138.117863	0.253688701	-1.97886883	5.00E-11	3.80E-08	carbohydrate (N-acetylglucosamino) sulfotransferase 7
Tyw3	326.8730387	461.406878	192.3391994	0.416853776	-1.262386691	6.38E-11	4.65E-08	tRNA-yW synthesizing protein 3 homolog (S. cerevisiae)
Osta	1154.047776	1803.655159	504.4403932	0.279676739	-1.838167826	8.02E-11	5.62E-08	NA
Hsp90aa1	4623.501767	6335.637107	2911.366428	0.459522283	-1.121793273	3.03E-10	1.94E-07	heat shock protein 90, alpha (cytosolic), class A member 1
Slco3a1	4771.815176	6424.679929	3118.950423	0.485463939	-1.042563962	3.08E-10	1.94E-07	solute carrier organic anion transporter family, member 3a1
B4galt5	4034.772252	6426.691967	1642.852536	0.255629575	-1.967873337	3.08E-10	1.94E-07	UDP-Gal:betaGlcNAc beta 1,4-galactosyltransferase, polypeptide 5
Frzb	341.4334484	492.0966049	190.7702918	0.387668376	-1.367105042	4.23E-10	2.57E-07	frizzled-related protein
Ccdc91	1049.424182	1408.580682	690.2676818	0.490044831	-1.029014358	6.58E-10	3.87E-07	coiled-coil domain containing 91
Mfsd2a	455.9409114	870.6068744	41.27494827	0.047409399	-4.3986831	1.68E-09	9.56E-07	major facilitator superfamily domain containing 2A
Abcb1a	266.7424288	161.0991726	372.385685	2.311530712	1.208848531	2.04E-09	1.13E-06	ATP-binding cassette, sub-family B (MDR/TAP), member 1A
Oxct1	21110.75281	27891.88304	14329.62258	0.513756011	-0.960844725	4.37E-09	2.34E-06	3-oxoacid CoA transferase 1
Gclm	3685.937386	4917.811895	2454.062877	0.499015198	-1.00284434	4.89E-09	2.55E-06	glutamate-cysteine ligase, modifier subunit
Tlcd2	93.12675788	138.8447953	47.40872044	0.341451189	-1.550248738	5.64E-09	2.84E-06	TLC domain containing 2
Gpc3	910.3671915	466.9962007	1353.738182	2.898820548	1.535466026	5.77E-09	2.84E-06	glypican 3
Agps	8510.957798	13114.91678	3906.998821	0.297904965	-1.747075927	6.44E-09	3.09E-06	alkylglycerone phosphate synthase
Tmco3	1158.986446	1615.991442	701.9814501	0.434396762	-1.202914745	7.18E-09	3.35E-06	transmembrane and coiled-coil domains 3
Csmd1	143.2379489	67.0715332	219.4043646	3.271199481	1.709819739	1.42E-08	6.32E-06	CUB and Sushi multiple domains 1
Ccl6	108.7293301	58.43198045	159.0266797	2.721569223	1.444438732	2.04E-08	8.83E-06	chemokine (C-C motif) ligand 6
Abca1	1973.974758	1367.337814	2580.611703	1.887325632	0.916343361	2.42E-08	1.01E-05	ATP-binding cassette, sub-family A (ABC1), member 1
Slc16a9	4049.367791	5348.542585	2750.192998	0.51419484	-0.959612963	2.45E-08	1.01E-05	solute carrier family 16 (monocarboxylic acid transporters), member 9
Atp11a	31129.32621	49223.52069	13035.13173	0.264815104	-1.916942687	2.97E-08	1.20E-05	ATPase, class VI, type 11A
Il34	349.2278072	538.5068446	159.9487698	0.297022724	-1.751354786	3.17E-08	1.26E-05	interleukin 34
3110045C21Rik	139.4391061	201.9987643	76.87944779	0.380593654	-1.393676589	3.36E-08	1.27E-05	RIKEN cDNA 3110045C21 gene
Ifrd2	1072.03048	1399.452911	744.608048	0.532070813	-0.910309829	5.17E-08	1.91E-05	interferon-related developmental regulator 2
Hist1h4h	827.6300654	1083.506139	571.7539916	0.527688742	-0.92224089	5.44E-08	1.91E-05	histone cluster 1, H4h
9030619P08Rik	167.0246702	285.6473123	48.40202807	0.169446818	-2.561095552	5.56E-08	1.91E-05	lymphocyte antigen 6 complex pseudogene
Ces2b	124.074298	217.895453	30.25314295	0.13884247	-2.848479157	5.57E-08	1.91E-05	carboxyesterase 2B
Cmya5	107.2566293	58.73552494	155.7777337	2.65218935	1.407183778	5.62E-08	1.91E-05	cardiomyopathy associated 5
Folh1	4661.950777	2967.207716	6356.693838	2.142315081	1.09917068	5.65E-08	1.91E-05	folate hydrolase 1
Hnf1b	5497.863626	7926.427145	3069.300106	0.387223657	-1.368761	7.63E-08	2.53E-05	HNF1 homeobox B
Serpinf2	5051.264284	6554.455073	3548.073495	0.541322422	-0.885439948	8.07E-08	2.63E-05	serine (or cysteine) peptidase inhibitor, clade F, member 2
Me1	5666.448198	7501.881267	3831.015128	0.510673922	-0.969525707	8.84E-08	2.83E-05	malic enzyme 1, NADP(+)-dependent, cytosolic
Mogat1	1209.531887	1664.688809	754.3749656	0.453162754	-1.141898805	1.01E-07	3.13E-05	monoacylglycerol O-acyltransferase 1
Dusp9	132.4164147	187.1092329	77.72359659	0.415391562	-1.267456183	1.19E-07	3.62E-05	dual specificity phosphatase 9
Pdk3	839.6521456	1165.472545	513.8317468	0.440878465	-1.181547085	1.24E-07	3.71E-05	pyruvate dehydrogenase kinase, isoenzyme 3
Susd3	807.1014649	1123.563156	490.6397734	0.436681971	-1.195345127	1.76E-07	5.03E-05	sushi domain containing 3
Ugt2b38	9559.119928	14143.37707	4974.862787	0.351745044	-1.507397999	1.77E-07	5.03E-05	UDP glucuronosyltransferase 2 family, polypeptide B38
Keg1	23262.02101	35302.45324	11221.58879	0.317869943	-1.653491491	1.81E-07	5.09E-05	kidney expressed gene 1
Pde3b	731.019101	510.2357347	951.8024673	1.865417105	0.899498252	2.25E-07	6.20E-05	phosphodiesterase 3B, cGMP-inhibited
Amacr	3263.207055	4695.102236	1831.311874	0.390047284	-1.358279069	2.31E-07	6.28E-05	alpha-methylacyl-CoA racemase
Spats2l	196.6967667	271.0803296	122.3132038	0.451206489	-1.148140278	2.52E-07	6.76E-05	spermatogenesis associated, serine-rich 2-like
Gm15348	134.5111778	244.9625243	24.05983135	0.098218417	-3.34786262	2.76E-07	7.29E-05	predicted gene 15348
Cyb561	590.4809432	770.5983821	410.3635043	0.53252578	-0.909076726	2.85E-07	7.29E-05	cytochrome b-561
0610012H03Rik	2115.195007	2779.840372	1450.549643	0.521810409	-0.93840237	2.88E-07	7.29E-05	NA
Col1a1	468.8216899	653.519914	284.1234658	0.434758696	-1.20171321	3.14E-07	7.83E-05	collagen, type I, alpha 1
Cpe	1435.848038	1913.805213	957.8908638	0.500516383	-0.998510801	3.19E-07	7.86E-05	carboxypeptidase E
Por	6093.837473	7757.699694	4429.975251	0.571042374	-0.80833029	3.58E-07	8.63E-05	P450 (cytochrome) oxidoreductase
Col3a1	682.6116583	960.2785711	404.9447455	0.421695076	-1.245727917	3.60E-07	8.63E-05	collagen, type III, alpha 1
Reep5	1640.390597	2096.747475	1184.033719	0.5647002	-0.824442952	3.81E-07	9.03E-05	receptor accessory protein 5
Vwa1	843.1194831	1253.627237	432.611729	0.34508801	-1.534963745	5.43E-07	0.000125709	von Willebrand factor A domain containing 1
Trp53inp2	4042.316494	5114.380801	2970.252187	0.58076477	-0.783974156	5.45E-07	0.000125709	transformation related protein 53 inducible nuclear protein 2
Cyp2c44	1206.31162	606.4549395	1806.168302	2.978239905	1.574459972	5.81E-07	0.000132334	cytochrome P450, family 2, subfamily c, polypeptide 44
Tm6sf2	87.77901623	49.17257058	126.3854619	2.570243133	1.361904838	5.99E-07	0.000134868	transmembrane 6 superfamily member 2
Slco1a1	4845.302737	8645.267048	1045.338426	0.120914533	-3.047940441	6.91E-07	0.000153527	solute carrier organic anion transporter family, member 1a1
Slc17a3	10096.07405	14228.3776	5963.770503	0.419146207	-1.254474521	7.01E-07	0.000153887	solute carrier family 17 (sodium phosphate), member 3
Cfi	209.7208055	103.2490545	316.1925564	3.062425685	1.614674835	7.39E-07	0.000160429	complement component factor i
Med21	392.7821482	514.8771682	270.6871281	0.525731465	-0.927602011	7.53E-07	0.00016154	mediator complex subunit 21
1600020E01Rik	132.8109757	79.64785103	185.9741003	2.334954402	1.223394376	7.63E-07	0.00016175	RIKEN cDNA 1600020E01 gene
Slc22a7	990.7003473	1867.631632	113.769063	0.060916222	-4.037029722	8.04E-07	0.000166915	solute carrier family 22 (organic anion transporter), member 7
Ces2c	1456.096444	1858.328646	1053.864241	0.567103264	-0.818316634	8.06E-07	0.000166915	carboxylesterase 2C
Ugt2b37	1055.211583	1662.156359	448.2668067	0.269689915	-1.890626523	8.32E-07	0.000170111	UDP glucuronosyltransferase 2 family, polypeptide B37
Trpv5	374.4750715	489.4899478	259.4601953	0.530062357	-0.915766006	8.40E-07	0.000170111	transient receptor potential cation channel, subfamily V, member 5
Gss	3443.645595	4384.364566	2502.926623	0.570875571	-0.808751768	9.06E-07	0.000181518	glutathione synthetase
Ttr	523.6744353	841.6540029	205.6948677	0.244393619	-2.032721477	9.17E-07	0.000181642	transthyretin
Fbp2	407.9388921	227.022911	588.8548731	2.593812538	1.375074216	9.76E-07	0.000189761	fructose bisphosphatase 2
Ly6a	4632.326129	6264.789802	2999.862457	0.47884487	-1.06236975	9.79E-07	0.000189761	lymphocyte antigen 6 complex, locus A
Kap	229817.0482	355923.0634	103711.033	0.291386099	-1.778996041	9.92E-07	0.000190373	kidney androgen regulated protein
Klk1	17499.28978	22407.3108	12591.26877	0.561926814	-0.83154585	1.04E-06	0.000196993	kallikrein 1
Greb1l	926.3306332	573.9790082	1278.682258	2.22775091	1.155587931	1.06E-06	0.000199975	growth regulation by estrogen in breast cancer-like
Cyp2d12	4665.513459	2781.89548	6549.131438	2.354197519	1.235235369	1.17E-06	0.000217505	cytochrome P450, family 2, subfamily d, polypeptide 12
Omd	102.1821643	58.60700151	145.7573271	2.487029251	1.314423476	1.27E-06	0.000233289	osteomodulin
Abhd2	2858.528049	3613.855083	2103.201015	0.581982666	-0.780951911	1.33E-06	0.000241482	abhydrolase domain containing 2
Acsm1	2058.074941	2733.022119	1383.127764	0.506079974	-0.982562707	1.34E-06	0.000241482	acyl-CoA synthetase medium-chain family member 1
Ace	4012.162438	5981.879689	2042.445187	0.341438694	-1.550301533	1.41E-06	0.000251357	angiotensin I converting enzyme (peptidyl-dipeptidase A) 1
Hyou1	3954.98318	4967.003903	2942.962457	0.592502546	-0.755106742	1.46E-06	0.000259083	hypoxia up-regulated 1
Cyp27b1	205.4584536	127.234647	283.6822602	2.229599146	1.156784355	1.56E-06	0.000273006	cytochrome P450, family 27, subfamily b, polypeptide 1
Itfg1	3242.428962	4074.901627	2409.956296	0.591414595	-0.757758248	1.62E-06	0.000281126	integrin alpha FG-GAP repeat containing 1
Vill	999.8229699	1378.313061	621.3328783	0.450792273	-1.149465307	1.69E-06	0.000291214	villin-like
Irf5	197.388327	267.4994621	127.2771919	0.475803543	-1.071562079	1.84E-06	0.000312999	interferon regulatory factor 5
Slc22a19	651.2322576	1199.919176	102.5453395	0.085460206	-3.548603402	1.92E-06	0.000323641	solute carrier family 22 (organic anion transporter), member 19
Ggt1	18490.60826	24213.83159	12767.38492	0.527276523	-0.923368333	2.01E-06	0.000335809	gamma-glutamyltransferase 1
Tnfsf10	1129.147094	1457.732604	800.5615844	0.549182739	-0.864641813	2.08E-06	0.00034408	tumor necrosis factor (ligand) superfamily, member 10
Slc39a14	906.14179	1171.9429	640.3406801	0.546392388	-0.871990709	2.27E-06	0.000371506	solute carrier family 39 (zinc transporter), member 14
Mvd	533.2378731	705.4839285	360.9918178	0.511693893	-0.96664708	2.29E-06	0.000371506	mevalonate (diphospho) decarboxylase
E2f3	154.7029177	209.1642756	100.2415598	0.479247995	-1.061155698	2.33E-06	0.000371506	E2F transcription factor 3
Slc9a8	2352.657487	3396.12276	1309.192215	0.38549614	-1.375211681	2.36E-06	0.000371506	solute carrier family 9 (sodium/hydrogen exchanger), member 8
Pigr	8614.099106	4784.673181	12443.52503	2.600705327	1.378902944	2.36E-06	0.000371506	polymeric immunoglobulin receptor
Nr1d2	2803.92678	3540.59702	2067.256539	0.583872304	-0.776275217	2.36E-06	0.000371506	nuclear receptor subfamily 1, group D, member 2
Arhgap15	221.5644675	137.3741233	305.7548116	2.225708921	1.154264929	2.43E-06	0.00037886	Rho GTPase activating protein 15
Heyl	130.9074338	180.8257451	80.98912253	0.44788491	-1.158800036	2.86E-06	0.000441484	hairy/enhancer-of-split related with YRPW motif-like
Hsp90ab1	22384.32936	27832.70072	16935.95801	0.608491363	-0.716691309	2.92E-06	0.000447807	heat shock protein 90 alpha (cytosolic), class B member 1
Isoc2b	171.7946262	229.352695	114.2365573	0.498082472	-1.005543452	3.02E-06	0.000451385	isochorismatase domain containing 2b
Degs2	719.9109988	1048.586632	391.2353652	0.373107336	-1.422337368	3.02E-06	0.000451385	delta(4)-desaturase, sphingolipid 2
Esm1	689.0471111	877.5815292	500.5126931	0.570331845	-0.810126505	3.02E-06	0.000451385	endothelial cell-specific molecule 1
Col4a1	7151.623489	8912.581897	5390.66508	0.604837649	-0.72538015	3.05E-06	0.000451385	collagen, type IV, alpha 1
Ier5	383.1039356	267.1675171	499.040354	1.867893071	0.901411869	3.35E-06	0.000492568	immediate early response 5
Zfp939	66.7424482	36.57419498	96.91070143	2.649701558	1.405829875	3.39E-06	0.000493537	zinc finger protein 939
BC048355	285.0608231	371.8471727	198.2744736	0.533214955	-0.90721085	3.56E-06	0.000514206	NA
Ctps	472.2705967	631.6423514	312.898842	0.495373436	-1.013411587	4.01E-06	0.000576123	cytidine 5'-triphosphate synthase
Hspa4l	1236.332266	1557.638258	915.026275	0.587444659	-0.767475148	4.10E-06	0.000583801	heat shock protein 4 like
P4ha1	1435.105213	1876.707135	993.5032912	0.52938643	-0.91760688	4.27E-06	0.000602896	procollagen-proline, 2-oxoglutarate 4-dioxygenase (proline 4-hydroxylase), alpha 1 polypeptide
Chst11	1714.580054	2416.696881	1012.463227	0.418945063	-1.255167022	4.31E-06	0.0006044	carbohydrate sulfotransferase 11
C1qtnf3	1657.371625	2355.718965	959.0242838	0.407104709	-1.296528186	4.99E-06	0.000694615	C1q and tumor necrosis factor related protein 3
Stk16	1160.63444	1451.123194	870.1456854	0.599635985	-0.737841132	5.11E-06	0.00070545	serine/threonine kinase 16
Slc22a2	4437.534968	5953.971688	2921.098247	0.490613392	-1.027341479	5.17E-06	0.000707782	solute carrier family 22 (organic cation transporter), member 2
Pltp	161.1726553	106.1051264	216.2401842	2.037980552	1.027140285	7.31E-06	0.000993771	phospholipid transfer protein
Sec11a	1260.762696	1580.664648	940.8607445	0.595231092	-0.748478207	7.41E-06	0.00100021	SEC11 homolog A, signal peptidase complex subunit
Gcnt2	1525.044713	1941.820311	1108.269114	0.570737213	-0.809101461	7.68E-06	0.001023769	glucosaminyl (N-acetyl) transferase 2, I-branching enzyme
N28178	34.8170296	13.88801726	55.74604194	4.013966925	2.005028729	7.80E-06	0.001023769	NA
Shisa7	220.8675707	152.1594781	289.5756632	1.903106311	0.928356156	7.81E-06	0.001023769	shisa family member 7
Hus1	1018.366882	1270.653917	766.0798467	0.602902046	-0.730004469	8.08E-06	0.00105182	HUS1 checkpoint clamp component
Tmem206	403.150533	511.9159018	294.3851643	0.575065481	-0.798201854	8.36E-06	0.001080538	transmembrane protein 206
Edn1	43.19585235	19.97869538	66.41300933	3.324191498	1.733003495	8.54E-06	0.001095862	endothelin 1
Dab1	106.7870009	68.09954987	145.4744519	2.136202841	1.095048643	9.05E-06	0.001147036	disabled 1
ORF63	549.4733685	798.3322839	300.6144531	0.376553046	-1.40907498	9.06E-06	0.001147036	NA
Abca3	16179.75101	20482.69084	11876.81117	0.579846235	-0.78625772	9.17E-06	0.001152907	ATP-binding cassette, sub-family A (ABC1), member 3

Tnfrsf12a	251.8152088	330.2468148	173.3836028	0.525012188	-0.92957718	9.58E-06	0.001196184	tumor necrosis factor receptor superfamily, member 12a
Mpv17l	6453.706937	9711.138287	3196.275588	0.329135009	-1.603248606	1.03E-05	0.001274313	Mpv17 transgene, kidney disease mutant-like
Sema4g	937.0295941	1171.33733	702.7218586	0.599931242	-0.737130932	1.06E-05	0.001309924	sema domain, immunoglobulin domain (Ig), transmembrane domain (TM) and short cytoplasmic domain, (semaphorin) 4G
Sepx1	2699.124502	3403.71237	1994.536634	0.585988596	-0.771055506	1.13E-05	0.001384615	NA
Pi4ka	4985.68973	6150.926292	3820.453167	0.621118346	-0.687059913	1.21E-05	0.001469428	phosphatidylinositol 4-kinase, catalytic, alpha polypeptide
Adamts1	1740.789903	2164.802724	1316.777082	0.608266549	-0.717224429	1.27E-05	0.00153251	a disintegrin-like and metallopeptidase (reprolysin type) with thrombospondin type 1 motif, 1
B3galt5	1014.425224	1342.15521	686.6952377	0.511636235	-0.966809654	1.29E-05	0.001546954	UDP-Gal:betaGlcNAc beta 1,3-galactosyltransferase, polypeptide 5
Kcnk5	3232.996195	4533.822325	1932.170066	0.426168016	-1.230505772	1.40E-05	0.001660121	potassium channel, subfamily K, member 5
1810046J19Rik	837.8070134	1047.809035	627.8049921	0.599159743	-0.738987401	1.46E-05	0.001707086	NA
Hbegf	280.425025	362.0289871	198.8210628	0.549185479	-0.864634614	1.46E-05	0.001707086	heparin-binding EGF-like growth factor
Slc22a12	22703.6449	27882.5029	17524.78689	0.628522732	-0.669963171	1.49E-05	0.001728265	solute carrier family 22 (organic anion/cation transporter), member 12
Spink8	141.4284491	187.8537327	95.0031656	0.505729454	-0.983562291	1.58E-05	0.001820596	serine peptidase inhibitor, Kazal type 8
Serpine2	406.6955245	296.3589387	517.0321103	1.744614529	0.80290831	1.61E-05	0.001846171	serine (or cysteine) peptidase inhibitor, clade E, member 2
Hspd1	6576.953316	8080.320883	5073.58575	0.627894093	-0.671406855	1.63E-05	0.001846171	heat shock protein 1 (chaperonin)
Asrgl1	198.4298361	117.5139202	279.345752	2.377129036	1.249220218	1.64E-05	0.001846171	asparaginase like 1
Slc26a6	123.678518	167.1205884	80.23644749	0.480111088	-1.058559841	1.64E-05	0.001846171	solute carrier family 26, member 6
Elovl1	1229.123672	1517.854424	940.3929194	0.619554092	-0.690697848	1.73E-05	0.001936028	elongation of very long chain fatty acids (FEN1/Elo2, SUR4/Elo3, yeast)-like 1
Cit	365.5173889	504.5804224	226.4543554	0.448797348	-1.155863944	1.77E-05	0.001955563	citron
1500011B03Rik	115.7388224	75.67614015	155.8015047	2.058792961	1.041798755	1.79E-05	0.001955563	RIKEN cDNA 1500011B03 gene
Pth1r	8167.099347	10118.28176	6215.916929	0.614325344	-0.702925191	1.79E-05	0.001955563	parathyroid hormone 1 receptor
Lap3	7353.283156	9530.588778	5175.977535	0.543091057	-0.880733988	1.79E-05	0.001955563	leucine aminopeptidase 3
Azin1	3223.87912	3971.389007	2476.369233	0.623552422	-0.681417243	1.93E-05	0.002088288	antizyme inhibitor 1
Unc13c	90.92146462	124.0548553	57.7880739	0.465826781	-1.102134511	1.95E-05	0.00209342	unc-13 homolog C (C. elegans)
Tpk1	1466.100395	1811.379508	1120.821283	0.618766679	-0.692532586	1.95E-05	0.00209342	thiamine pyrophosphokinase
Fam55c	704.2309412	959.1181373	449.3437451	0.468496766	-1.093889007	2.01E-05	0.002145345	NA
Bdh1	1553.459217	2223.379516	883.5389175	0.397385562	-1.33138864	2.03E-05	0.002150559	3-hydroxybutyrate dehydrogenase, type 1
Ect2	70.39603625	100.5323734	40.25969909	0.400465022	-1.320251858	2.05E-05	0.002154306	ect2 oncogene
Pcyox1l	142.5171427	188.360256	96.67402951	0.513240062	-0.962294307	2.11E-05	0.002204969	prenylcysteine oxidase 1 like
Paqr9	1903.758528	2345.795918	1461.721138	0.623123745	-0.682409401	2.13E-05	0.002217337	progestin and adipoQ receptor family member IX
Hes6	676.5940737	846.8999367	506.2882107	0.597813495	-0.742232631	2.20E-05	0.00227581	hairy and enhancer of split 6
Prr5l	125.3050239	82.56340962	168.0466382	2.035364564	1.025287226	2.32E-05	0.002383798	proline rich 5 like
Sh3bp2	363.4554178	585.8884306	141.0224051	0.240698395	-2.054701571	2.54E-05	0.00259676	SH3-domain binding protein 2
Tmem189	2598.9031	3318.327251	1879.478949	0.566393489	-0.820123414	2.56E-05	0.002601825	transmembrane protein 189
Prr15l	1065.563023	1318.244143	812.8819035	0.616639875	-0.697499911	2.60E-05	0.002609439	proline rich 15-like
Tgm1	761.415801	1145.646233	377.1853688	0.329233718	-1.602816	2.60E-05	0.002609439	transglutaminase 1, K polypeptide
D16H22S680E	896.1089835	1113.214034	679.0039332	0.609949132	-0.713239165	2.61E-05	0.002609439	NA
Glb1	2367.327706	3054.350023	1680.30539	0.550135177	-0.86214194	2.71E-05	0.0026955	galactosidase, beta 1
Apln	154.2499316	204.8797966	103.6200665	0.505760296	-0.983474311	2.72E-05	0.002697609	apelin
AI464131	1115.552696	1380.205274	850.9001183	0.616502584	-0.697821153	2.84E-05	0.00279287	expressed sequence AI464131
Atp2a3	416.9372408	305.8941168	527.9803647	1.726023273	0.787451917	2.87E-05	0.002807334	ATPase, Ca++ transporting, ubiquitous
Tmem176b	8620.215414	10682.28941	6558.141422	0.613926582	-0.703861957	2.92E-05	0.002814768	transmembrane protein 176B
4931417G12Rik	39.12046329	58.38181851	19.85910806	0.340159121	-1.555718322	3.01E-05	0.002884429	NA
Cxcl12	2958.39882	2287.040471	3629.757169	1.587097918	0.66639114	3.36E-05	0.003201515	chemokine (C-X-C motif) ligand 12
Slc22a26	280.309096	473.2353009	87.38289109	0.184649985	-2.437134952	3.40E-05	0.003222796	solute carrier family 22 (organic cation transporter), member 26
Paqr7	1429.841468	1893.797912	965.885025	0.510025393	-0.971359016	3.42E-05	0.003231946	progestin and adipoQ receptor family member VII
1700021F05Rik	409.9438388	513.5128163	306.3748613	0.59662554	-0.745102358	3.46E-05	0.003246927	RIKEN cDNA 1700021F05 gene
Cadm2	33.72991567	15.85224457	51.60758677	3.255538138	1.70289604	3.47E-05	0.003246927	cell adhesion molecule 2
Bcl6b	122.7173387	163.1976277	82.23704959	0.503910815	-0.988759675	3.59E-05	0.003340934	B cell CLL/lymphoma 6, member B
Mep1b	1781.536439	2895.396271	667.6766077	0.230599388	-2.116539412	3.65E-05	0.003375908	meprin 1 beta
Arpp19	1829.832928	2238.946887	1420.71897	0.634547867	-0.656199098	3.67E-05	0.003375908	cAMP-regulated phosphoprotein 19
Mat2a	7669.189865	9683.749771	5654.629959	0.583929789	-0.776133184	3.72E-05	0.003404722	methionine adenosyltransferase II, alpha
Sypl2	1485.081658	1970.038191	1000.125125	0.507667887	-0.978043092	3.79E-05	0.003454743	synaptophysin-like 2
Aldh1a7	772.0700961	322.146791	1221.993401	3.793281309	1.923446366	3.87E-05	0.003504825	aldehyde dehydrogenase family 1, subfamily A7
Cd36	7037.545171	9394.163676	4680.926665	0.498280297	-1.004970567	3.90E-05	0.003508911	CD36 antigen
Sepp1	40118.41294	52829.30799	27407.5179	0.518793809	-0.946766832	3.91E-05	0.003508911	selenoprotein P, plasma, 1
Wisp1	128.4619998	186.8632263	70.06077326	0.37493077	-1.415303866	4.02E-05	0.003573104	WNT1 inducible signaling pathway protein 1
Syt12	170.5026974	223.7390087	117.266386	0.524121326	-0.932027282	4.07E-05	0.003602847	synaptotagmin XII
Cmtm6	4216.237655	5540.695488	2891.779822	0.521916396	-0.938109369	4.21E-05	0.003704883	CKLF-like MARVEL transmembrane domain containing 6
1300002K09Rik	258.0777061	354.2952363	161.8601759	0.456851121	-1.130204	4.25E-05	0.003724205	NA
Appl2	823.0743219	626.8281529	1019.320491	1.626156206	0.701465847	4.29E-05	0.003737425	adaptor protein, phosphotyrosine interaction, PH domain and leucine zipper containing 2
2610528J11Rik	910.6790961	1122.474235	698.8839573	0.622628062	-0.683557495	4.36E-05	0.003785022	RIKEN cDNA 2610528J11 gene
Metrnl	313.7406059	395.6555676	231.8256443	0.585927921	-0.771204894	4.45E-05	0.003844853	meteorin, glial cell differentiation regulator-like
Trim47	489.7604103	614.2442041	365.2766166	0.594676538	-0.749822938	4.57E-05	0.003925186	tripartite motif-containing 47
Dnaja4	539.3559941	685.3703558	393.3416323	0.573911067	-0.8011009	4.65E-05	0.003975201	DnaJ heat shock protein family (Hsp40) member A4
Ccrn4l	277.4183276	358.1259951	196.71066	0.549277804	-0.864392101	4.77E-05	0.004059974	NA
Rnf24	1118.723523	1888.233832	349.213215	0.184941721	-2.434857374	5.13E-05	0.004351902	ring finger protein 24
Stra6	90.65710099	24.09860393	157.2155981	6.523846713	2.705722884	5.24E-05	0.004424011	stimulated by retinoic acid gene 6
Tmem176a	6463.348372	8135.844751	4790.851994	0.588857352	-0.764009905	5.30E-05	0.00445506	transmembrane protein 176A
Rhobtb1	1804.771576	1079.577714	2529.965438	2.343476904	1.228650576	5.40E-05	0.004515686	Rho-related BTB domain containing 1
Skiv2l2	1792.43836	2189.826914	1395.049805	0.637059393	-0.650500213	5.72E-05	0.00475938	superkiller viralicidic activity 2-like 2 (S. cerevisiae)
Slc41a2	539.0418166	670.9103369	407.1732963	0.60689674	-0.720477024	5.77E-05	0.00477951	solute carrier family 41, member 2
Irf2bp2	2745.96667	2135.906448	3356.026891	1.571242455	0.651905817	5.90E-05	0.004841295	interferon regulatory factor 2 binding protein 2
Prss8	4588.743645	5578.395028	3599.092262	0.645184187	-0.632217015	5.92E-05	0.004841295	protease, serine 8 (prostasin)
Uros	317.2630056	398.3046076	236.2214037	0.593067213	-0.753732478	5.97E-05	0.00485327	uroporphyrinogen III synthase
Eci3	2332.752119	2960.001435	1705.502803	0.5761831	-0.795400749	6.22E-05	0.005026831	enoyl-Coenzyme A delta isomerase 3
Aldh1a1	960.7937972	158.1788624	1763.408732	11.14819455	3.47873818	6.23E-05	0.005026831	aldehyde dehydrogenase family 1, subfamily A1
Gm1653	36.43608021	58.91804758	13.95411284	0.236839363	-2.078019218	6.28E-05	0.005041226	predicted gene 1653
Csf1r	804.3075935	1075.633879	532.9813078	0.495504389	-1.013030259	6.43E-05	0.005137377	colony stimulating factor 1 receptor
Tnfrsf21	8858.253452	10732.57282	6983.934079	0.650723195	-0.619884115	6.62E-05	0.005242626	tumor necrosis factor receptor superfamily, member 21
Akr1c12	121.5486974	49.41890103	193.6784938	3.919117782	1.970528931	6.93E-05	0.005463693	aldo-keto reductase family 1, member C12
Nudt8	432.5067342	537.1808594	327.8326089	0.610283489	-0.712448536	7.17E-05	0.005607755	nudix (nucleoside diphosphate linked moiety X)-type motif 8
Fbxo36	336.9514435	420.968647	252.93424	0.600838665	-0.734950438	7.29E-05	0.005679015	F-box protein 36
Pcdhga2	56.14083916	32.63382763	79.64785068	2.440653042	1.287267219	7.36E-05	0.005704995	protocadherin gamma subfamily A, 2
Tuba1c	516.4402804	638.8413928	394.039168	0.616802813	-0.697118751	7.39E-05	0.005704995	tubulin, alpha 1C
Ift122	1096.660467	1336.55178	856.7691552	0.641029527	-0.641537283	7.71E-05	0.00593063	intraflagellar transport 122
Lipo1	580.8042738	856.9631969	304.6453507	0.355494089	-1.492102525	7.83E-05	0.00599674	lipase, member O1
Tsga10	156.1491914	203.1024255	109.1959573	0.537639849	-0.895288024	7.93E-05	0.006050364	testis specific 10
Agmo	150.8208492	85.06489379	216.5768047	2.546018634	1.348242978	8.05E-05	0.006099438	alkylglycerol monooxygenase
Bcl3	165.0377071	221.5434577	108.5319565	0.489890145	-1.029469827	8.07E-05	0.006099438	B cell leukemia/lymphoma 3
Lman2l	709.9282188	871.2369362	548.6195013	0.629701839	-0.667259213	8.20E-05	0.006161178	lectin, mannose-binding 2-like
Calb1	7194.857543	9090.267607	5299.447479	0.58298036	-0.778480815	8.22E-05	0.006161178	calbindin 1
Gsta3	2444.347884	1887.650758	3001.04501	1.589830639	0.668873087	8.25E-05	0.006162403	glutathione S-transferase, alpha 3
Tubb2c	2918.550015	3548.466814	2288.633216	0.644963962	-0.632709544	8.36E-05	0.00620236	NA
4931406C07Rik	16911.38965	20409.7837	13412.99559	0.657184603	-0.605629416	8.38E-05	0.00620236	RIKEN cDNA 4931406C07 gene
Ubiad1	1215.4349	1689.341638	741.5281615	0.438945057	-1.187887727	8.41E-05	0.00620236	UbiA prenyltransferase domain containing 1
Slc1a4	595.0721137	820.49698	369.6472475	0.45051628	-1.150348853	8.44E-05	0.00620236	solute carrier family 1 (glutamate/neutral amino acid transporter), member 4
Slc25a19	739.9943558	941.6231538	538.3655579	0.571742056	-0.80656368	8.65E-05	0.006334038	solute carrier family 25 (mitochondrial thiamine pyrophosphate carrier), member 19
Ahsa1	1199.928683	1464.933791	934.9235745	0.638201931	-0.647915121	8.91E-05	0.006496541	AHA1, activator of heat shock protein ATPase 1
Crtap	646.0592547	790.7346318	501.3838777	0.634073503	-0.657278006	9.04E-05	0.006562657	cartilage associated protein
Arfgap3	533.9983504	663.4885826	404.5081181	0.609668544	-0.713902983	9.42E-05	0.006783861	ADP-ribosylation factor GTPase activating protein 3
Flrt3	57.86249503	81.31258003	34.41241002	0.42321139	-1.240549639	9.53E-05	0.006837123	fibronectin leucine rich transmembrane protein 3
Cpeb3	1150.637187	1414.811643	886.4627311	0.626558832	-0.674478114	9.85E-05	0.007011274	cytoplasmic polyadenylation element binding protein 3
Rtkn	513.744232	637.6630565	389.8254074	0.611334471	-0.709966177	9.90E-05	0.007016805	rhotekin
Ttbk1	240.4814426	375.412858	105.5500273	0.281157198	-1.830551113	0.000100275	0.007082971	tau tubulin kinase 1
Kynu	558.0532424	185.5578582	930.5486266	5.014870486	2.326212442	0.000101809	0.007162397	kynureninase (L-kynurenine hydrolase)
Sbk1	1365.842845	1663.179789	1068.505901	0.642447623	-0.638349256	0.000102185	0.007162397	SH3-binding kinase 1
Slc16a4	1242.526296	1624.720903	860.3316877	0.529525832	-0.91722703	0.000102951	0.007188404	solute carrier family 16 (monocarboxylic acid transporters), member 4
Mlec	18311.64577	22946.21525	13677.07629	0.596049333	-0.746496353	0.00010374	0.007215888	malectin
Slc22a6	18539.68669	24209.06565	12870.30772	0.531631741	-0.911500851	0.00010456	0.007245268	solute carrier family 22 (organic anion transporter), member 6
Abhd3	1333.900196	1618.223373	1049.577019	0.648598356	-0.624602728	0.000105663	0.007293942	abhydrolase domain containing 3
Pepd	3523.953259	4335.917513	2711.989004	0.625470617	-0.676985982	0.000106161	0.007300703	peptidase D
Ntn1	2557.714521	3142.516204	1972.912839	0.6278131	-0.671592963	0.000109344	0.007472188	netrin 1
Chst3	173.6118258	222.1063641	125.1172875	0.563321488	-0.827969591	0.000109475	0.007472188	carbohydrate (chondroitin 6/keratan) sulfotransferase 3
Lasp1	2765.029459	3332.934107	2197.12481	0.659216396	-0.60117597	0.000114968	0.007812955	LIM and SH3 protein 1
Gmpr	909.7493914	1137.21872	682.2800631	0.599955005	-0.737073787	0.000115325	0.007812955	guanosine monophosphate reductase
Hspe1	1862.511392	2322.85864	1402.164144	0.603637311	-0.728246112	0.000120665	0.008144447	heat shock protein 1 (chaperonin 10)
Ptplb	1055.849689	1286.705027	824.9943503	0.641168203	-0.641225214	0.00012261	0.008245195	NA
Alb	221.8501786	134.4643381	309.2360191	2.299762327	1.201484771	0.00012503	0.008377019	albumin
Acnat2	3678.686121	2615.203815	4742.168428	1.813307399	0.858623518	0.000125861	0.008401811	acyl-coenzyme A amino acid N-acyltransferase 2
B4galt1	1729.434182	2173.927942	1284.940422	0.591068543	-0.758602653	0.00013117	0.008724224	UDP-Gal:betaGlcNAc beta 1,4- galactosyltransferase, polypeptide 1
Il13ra1	1335.52164	1634.973421	1036.069859	0.633692172	-0.6581459	0.000132198	0.008760657	interleukin 13 receptor, alpha 1
Chid1	523.8851914	643.5685234	404.2018594	0.628063438	-0.671017808	0.000133401	0.008797907	chitinase domain containing 1
Txndc16	422.5239804	322.9608524	522.0871084	1.616564684	0.692931235	0.000134205	0.008797907	thioredoxin domain containing 16
2410004N09Rik	91.14815053	57.06300492	125.2332961	2.194649516	1.133990561	0.000134209	0.008797907	NA
Akap7	386.3230893	294.3594631	478.2867155	1.624838931	0.700296712	0.000141477	0.009241132	A kinase (PRKA) anchor protein 7
Sox17	169.766815	120.7010195	218.8326105	1.813013771	0.858389883	0.000142798	0.009294131	SRY (sex determining region Y)-box 17
Zfp385a	176.2427604	126.9132054	225.5723154	1.777374661	0.829747825	0.000145361	0.009427266	zinc finger protein 385A
Nhp2	482.8967744	612.7852672	353.0082816	0.576071751	-0.795679582	0.000146392	0.009460453	NHP2 ribonucleoprotein
Zfhx2	484.0757166	594.206469	373.9449641	0.629318231	-0.668138356	0.000146934	0.009461924	zinc finger homeobox 2
Slc38a4	108.2940874	45.46333007	171.1248448	3.764019146	1.912273966	0.000147876	0.009489048	solute carrier family 38, member 4
Ggct	467.5391631	623.4952072	311.5831191	0.49973619	-1.000761396	0.000148488	0.009494903	gamma-glutamyl cyclotransferase
0610010O12Rik	1438.857347	1740.391498	1137.323196	0.653486987	-0.613769587	0.000150506	0.009590285	NA
Cdc42se1	2266.620039	2750.306721	1782.933357	0.648267098	-0.625339742	0.000152048	0.009654795	CDC42 small effector 1
Plcd4	34.94048208	51.25696192	18.62400223	0.363345808	-1.460584835	0.000153123	0.009655753	phospholipase C, delta 4
Erp29	1199.505167	1452.178817	946.8315174	0.652007526	-0.617039479	0.000158686	0.009972024	endoplasmic reticulum protein 29
Ghr	14153.8717	19019.92638	9287.817017	0.488320345	-1.034100208	0.000160584	0.010056657	growth hormone receptor
Hunk	444.1530571	579.0961502	309.209964	0.533952719	-0.905216096	0.000162099	0.010116773	hormonally upregulated Neu-associated kinase
Lrig2	856.1857833	665.5765556	1046.795011	1.572764248	0.653302432	0.000168266	0.010465808	leucine-rich repeats and immunoglobulin-like domains 2
Tstd1	327.3442194	245.6284427	409.0599962	1.665360867	0.735834829	0.000175972	0.010907864	thiosulfate sulfurtransferase (rhodanese)-like domain containing 1
Larp4b	3087.158547	3746.941296	2427.375797	0.647828617	-0.626315896	0.000184992	0.011389485	La ribonucleoprotein domain family, member 4B
Gm6300	1031.712406	1810.772015	252.652797	0.139527668	-2.841376859	0.000186672	0.011454257	solute carrier family 7 (cationic amino acid transporter, y+ system), member pseudogene
Inpp5j	627.5263614	766.9993155	488.0534074	0.63631531	-0.652186261	0.000191867	0.011729352	inositol polyphosphate 5-phosphatase J
Npas2	391.6035037	98.69830068	684.5087067	6.935364661	2.793971741	0.000192443	0.011729352	neuronal PAS domain protein 2
Samd9l	6946.464512	8768.228806	5124.700217	0.584462419	-0.774817833	0.000200454	0.012136432	sterile alpha motif domain containing 9-like
Rangap1	893.4232897	1089.66889	697.1776898	0.639806914	-0.644291512	0.000201804	0.012177756	RAN GTPase activating protein 1

Erp44	1400.650878	1683.599554	1117.702202	0.663876513	-0.591013182	0.000205295	0.01234751	endoplasmic reticulum protein 44
Arhgap27	844.9448239	1025.495273	664.3943748	0.647876584	-0.626209078	0.000210485	0.012618	Rho GTPase activating protein 27
Nceh1	5983.652374	7272.518348	4694.7864	0.645551675	-0.631395511	0.000213047	0.012713588	neutral cholesterol ester hydrolase 1
Slc6a15	586.8347464	752.8293716	420.8401212	0.559011294	-0.839050664	0.000213474	0.012713588	solute carrier family 6 (neurotransmitter transporter), member 15
Myo5a	1292.212669	1905.85475	678.5705878	0.35604528	-1.489867368	0.000215415	0.012787361	myosin VA
Pecr	4893.296651	6549.357367	3237.235936	0.494282989	-1.016590838	0.000221821	0.013124906	peroxisomal trans-2-enoyl-CoA reductase
Pex11c	185.5629571	234.3946809	136.7312333	0.583337612	-0.777596998	0.000224589	0.013245688	NA
Net1	3125.075689	2494.828761	3755.322617	1.505242634	0.589996058	0.000226743	0.01332954	neuroepithelial cell transforming gene 1
Adh1	6266.063714	8783.945948	3748.18148	0.426708168	-1.228678369	0.000232554	0.013627202	alcohol dehydrogenase 1 (class I)
Rarres2	341.5768394	259.1586988	423.9949799	1.636043791	0.710211364	0.000240799	0.014065156	retinoic acid receptor responder (tazarotene induced) 2
Mcfd2	2176.682606	2613.042117	1740.323095	0.666014177	-0.586375208	0.000241587	0.014066057	multiple coagulation factor deficiency 2
Ido2	212.0145036	266.0877673	157.94124	0.593568211	-0.752514265	0.000243081	0.014107974	indoleamine 2,3-dioxygenase 2
Creg1	1555.793959	1223.95272	1887.635197	1.542245192	0.625032148	0.000247673	0.014328858	cellular repressor of E1A-stimulated genes 1
Selenbp2	261.3587975	362.7334165	159.9841784	0.441051668	-1.180980421	0.000249583	0.01439368	selenium binding protein 2
Slc6a9	442.3047613	563.6714775	320.9380451	0.569370738	-0.812559746	0.000253065	0.014548424	solute carrier family 6 (neurotransmitter transporter, glycine), member 9
1500001M20Rik	200.035915	147.5296845	252.5421456	1.711805638	0.775518904	0.000257049	0.014676071	NA
Far1	5554.290877	6753.663138	4354.918616	0.644823191	-0.633024464	0.000257701	0.014676071	fatty acyl CoA reductase 1
C3	948.0470663	509.9184193	1386.175713	2.71842644	1.44277179	0.000264261	0.014933814	complement component 3
Trpv4	2272.244693	2789.342376	1755.147011	0.629233265	-0.668333152	0.000264685	0.014933814	transient receptor potential cation channel, subfamily V, member 4
St6galnac2	2262.25973	2720.63259	1803.88687	0.663039499	-0.592833276	0.000269071	0.015134434	ST6 (alpha-N-acetyl-neuraminyl-2,3-beta-galactosyl-1,3)-N-acetylgalactosaminide alpha-2,6-sialyltransferase 2
Mlx	797.6163915	963.8646073	631.3681756	0.65503824	-0.610348965	0.00027075	0.015135413	MAX-like protein X
H2-Gs10	473.6235386	355.1616088	592.0854684	1.667087472	0.737329804	0.000274108	0.015276261	NA
Thsd4	270.0162688	180.9718937	359.0606439	1.984068557	0.988461877	0.000276272	0.015349963	thrombospondin, type I, domain containing 4
Tmem117	250.493271	312.7189917	188.2675504	0.602034272	-0.732082478	0.000278664	0.015395695	transmembrane protein 117
Cep57l1	149.0824976	192.8586662	105.306329	0.546028504	-0.872951829	0.000278785	0.015395695	centrosomal protein 57-like 1
Lpar3	474.7322899	578.1773073	371.2872725	0.642168532	-0.638976125	0.000286207	0.015700464	lysophosphatidic acid receptor 3
Ddr1	2704.61984	3251.305182	2157.934498	0.663713302	-0.591367907	0.000286918	0.015700464	discoidin domain receptor family, member 1
Cyp2a5	5189.929816	2801.451398	7578.408234	2.705172126	1.435720393	0.000287251	0.015700464	cytochrome P450, family 2, subfamily a, polypeptide 5
Osmr	610.0434573	477.5428854	742.5440293	1.554926378	0.636846274	0.00028775	0.015700464	oncostatin M receptor
Tiam2	253.5834741	180.7166169	326.4503312	1.806421217	0.853134337	0.000291183	0.015840352	T cell lymphoma invasion and metastasis 2
Apoe	4325.699781	1711.747974	6939.651588	4.054131621	2.019392928	0.000292683	0.015874584	apolipoprotein E
Lass5	548.8497624	670.6007051	427.0988198	0.636889906	-0.650884088	0.000295906	0.015954437	NA
Abcc4	3347.513235	1625.778876	5069.247594	3.118042478	1.640640582	0.000305192	0.016389317	ATP-binding cassette, sub-family C (CFTR/MRP), member 4
Fuca2	7474.010638	9397.759563	5550.261713	0.590594138	-0.759761057	0.000305771	0.016389317	fucosidase, alpha-L- 2, plasma
Rtn4ip1	842.0042294	1013.710772	670.2976865	0.661231689	-0.596772228	0.000306779	0.016395153	reticulon 4 interacting protein 1
Slc33a1	2832.543334	3453.672527	2211.414141	0.640307998	-0.643162064	0.000315652	0.016770951	solute carrier family 33 (acetyl-CoA transporter), member 1
Lgr4	7884.658124	6059.091611	9710.224636	1.602587526	0.680403153	0.000329921	0.017427505	leucine-rich repeat-containing G protein-coupled receptor 4
Idi1	261.603672	338.109349	185.0979951	0.547450095	-0.86920064	0.000343842	0.018058133	isopentenyl-diphosphate delta isomerase
Stip1	1578.285909	1946.134736	1210.437083	0.621969826	-0.685083503	0.000350491	0.018354463	stress-induced phosphoprotein 1
Lpl	20645.33319	28734.98171	12555.68466	0.436947717	-1.19446743	0.000356604	0.018621085	lipoprotein lipase
Tmem125	312.1042444	390.0454021	234.1630868	0.600348281	-0.736128398	0.000363923	0.018948941	transmembrane protein 125
Zfp830	575.9157169	698.4877064	453.3437275	0.649036087	-0.6236294	0.00038076	0.019546386	zinc finger protein 830
H2afj	1392.707244	1672.400161	1113.014327	0.665519146	-0.587447924	0.000382283	0.019569449	H2A histone family, member J
Myc	222.841133	118.0490416	327.6332245	2.775399276	1.472695336	0.00038681	0.019745733	myelocytomatosis oncogene
Ldhd	13854.89265	18037.83583	9671.949478	0.536203432	-0.899147641	0.000390075	0.019837362	lactate dehydrogenase D
Them4	482.8658422	587.9260691	377.8056154	0.642607354	-0.637990605	0.000397893	0.020142209	thioesterase superfamily member 4
Aplnr	77.35480062	110.4422305	44.26737072	0.400819238	-1.318976339	0.000405315	0.020461103	apelin receptor
Sec23b	1876.691001	2365.964079	1387.417924	0.586407011	-0.770025742	0.000407143	0.02049662	SEC23 homolog B, COPII coat complex component
Dcaf12l1	72.8214784	96.91086411	48.73209269	0.502854795	-0.99178623	0.000413545	0.020761565	DDB1 and CUL4 associated factor 12-like 1
Gatsl3	150.9923645	198.7430677	103.2416612	0.519473018	-0.944879278	0.000421408	0.021040397	GATS protein-like 3
Rdh16	3666.343384	2767.638763	4565.048006	1.649437805	0.72197438	0.000431145	0.021409235	retinol dehydrogenase 16
Pdgfd	257.7065634	317.5010728	197.912054	0.623342946	-0.681901983	0.000448472	0.022089053	platelet-derived growth factor, D polypeptide
Rabggta	916.593755	1100.798684	732.3888263	0.665324947	-0.587868964	0.000451255	0.022166238	Rab geranylgeranyl transferase, a subunit
Slc1a2	428.6885479	335.2414183	522.1356775	1.557491554	0.63922434	0.000457199	0.02239633	solute carrier family 1 (glial high affinity glutamate transporter), member 2
Npdc1	295.7576372	365.5675372	225.9477371	0.618073855	-0.694148855	0.000459626	0.02239633	neural proliferation, differentiation and control 1
Cml1	3371.558004	4527.95395	2215.162057	0.489219211	-1.031447038	0.000467707	0.02272932	NA
Znrf2	2242.911051	2729.054194	1756.767908	0.643727747	-0.635477439	0.000472563	0.022834631	zinc and ring finger 2
Mttp	1040.98426	787.228404	1294.740115	1.644681656	0.717808363	0.000473633	0.022834631	microsomal triglyceride transfer protein
Cyp51	2451.643438	3589.735247	1313.551629	0.365918804	-1.450404538	0.000475521	0.02286517	cytochrome P450, family 51
Irf6	1169.190606	1428.454589	909.9266238	0.637000736	-0.650633057	0.000482701	0.023149341	interferon regulatory factor 6
Akr1c14	2832.248952	4539.55951	1124.938393	0.24780783	-2.012706319	0.000506244	0.024088224	aldo-keto reductase family 1, member C14
Ugt3a2	20048.42831	24097.54896	15999.30765	0.663939211	-0.590876937	0.000513694	0.024325597	UDP glycosyltransferases 3 family, polypeptide A2
Fam163a	266.2571402	358.4171284	174.097152	0.485738929	-1.041746982	0.000515661	0.024325597	family with sequence similarity 163, member A
Plod2	741.822528	931.1015514	552.5435045	0.593429904	-0.752850465	0.000516572	0.024325597	procollagen lysine, 2-oxoglutarate 5-dioxygenase 2
BC026585	3235.943893	3917.315526	2554.57226	0.652123181	-0.616783592	0.000529924	0.024890035	cDNA sequence BC026585
Als2cr4	274.5854476	350.259796	198.9110992	0.567895892	-0.816301619	0.00053855	0.025230189	NA
Slc46a1	481.4747571	579.6382438	383.3112705	0.661293962	-0.596636367	0.00055958	0.026148154	solute carrier family 46, member 1
Pnrc1	1746.855922	1236.751768	2256.960076	1.824909521	0.867824937	0.000573291	0.026652193	proline-rich nuclear receptor coactivator 1
C8a	247.3954793	391.5251895	103.265769	0.263752555	-1.922743026	0.000592151	0.027389231	complement component 8, alpha polypeptide
Mrc1	165.3321949	119.7224818	210.941908	1.761923949	0.817151654	0.000638079	0.029364503	mannose receptor, C type 1
Hoxa9	373.4557315	453.5076903	293.4037726	0.646965374	-0.628239594	0.000670519	0.030702339	homeobox A9
Ptbp2	624.9588475	475.6374013	774.2802936	1.627879329	0.70299376	0.000674721	0.030817327	polypyrimidine tract binding protein 2
Olfml1	268.9659763	198.1422135	339.7897392	1.714878083	0.778106013	0.00070121	0.031867442	olfactomedin-like 1
Mettl7b	3295.641975	4185.978624	2405.305326	0.574610036	-0.799344905	0.000707546	0.032075398	methyltransferase like 7B
Akr1c18	41.64818972	81.86901781	1.427361639	0.017434698	-5.841894778	0.000714477	0.032309272	aldo-keto reductase family 1, member C18
Gm129	43.62329726	66.96661275	20.27998177	0.302837204	-1.723385641	0.000721452	0.032463555	NA
Gclc	7961.514671	10959.17607	4963.853269	0.45294037	-1.142606963	0.000728524	0.032663101	glutamate-cysteine ligase, catalytic subunit
Hsf2bp	141.2802518	190.0707947	92.48970894	0.48660663	-1.039172117	0.000730745	0.032663101	heat shock transcription factor 2 binding protein
2610029I01Rik	163.8776565	204.8137017	122.9416113	0.600260677	-0.736338934	0.000731263	0.032663101	NA
Fmo4	798.9575959	574.9533171	1022.961875	1.77920858	0.83123565	0.000743104	0.033011375	flavin containing monooxygenase 4
Aacs	2556.180392	3875.899964	1236.460819	0.319012573	-1.648314811	0.000744495	0.033011375	acetoacetyl-CoA synthetase
Rasd2	86.1739618	112.5420249	59.80589866	0.531409477	-0.912104137	0.00075941	0.033428731	RASD family, member 2
Cntln	318.1122466	245.6729301	390.5515631	1.589721598	0.668774134	0.000771682	0.033805601	centlein, centrosomal protein
Dnaja1	1466.453336	1886.54738	1046.359292	0.554642466	-0.850370014	0.00077388	0.033820606	DnaJ heat shock protein family (Hsp40) member A1
Entpd4	5521.800597	7127.267472	3916.333722	0.549486004	-0.863845361	0.00081625	0.035485018	ectonucleoside triphosphate diphosphohydrolase 4
Apeh	2930.337945	3558.065431	2302.610459	0.647152365	-0.627822676	0.000817807	0.035485018	acylpeptide hydrolase
Ftcd	536.7460303	421.2983136	652.193747	1.548056866	0.630458468	0.000821022	0.035539931	formiminotransferase cyclodeaminase
Nat2	357.6191766	436.2619124	278.9764409	0.639470082	-0.645051231	0.000832019	0.035761127	N-acetyltransferase 2 (arylamine N-acetyltransferase)
Olfr1033	114.4225021	149.0127737	79.83223055	0.535740853	-0.900392781	0.00083585	0.035841265	olfactory receptor 1033
Aspdh	954.9335947	1201.192912	708.6742771	0.589975407	-0.761273278	0.000848952	0.036309197	aspartate dehydrogenase domain containing
Gal3st1	266.8021892	325.5311295	208.0732489	0.639180803	-0.645704014	0.000850748	0.036309197	galactose-3-O-sulfotransferase 1
Apob	22488.65673	14108.52874	30868.78473	2.187952076	1.129581138	0.000866427	0.036828289	apolipoprotein B
Mylip	721.8990438	556.2058141	887.5922735	1.595798266	0.674278283	0.000866952	0.036828289	myosin regulatory light chain interacting protein
Cyp2j11	3570.518671	4357.798203	2783.239138	0.638680134	-0.646834519	0.00087	0.036871799	cytochrome P450, family 2, subfamily j, polypeptide 11
Dhrs7	190.2593048	144.4825745	236.0360351	1.633664378	0.708111624	0.000884162	0.037298523	dehydrogenase/reductase (SDR family) member 7
Kif20b	296.9934852	457.9048148	136.0821556	0.297184374	-1.750569835	0.000886735	0.037320673	kinesin family member 20B
Fabp1	31.84839592	7.323028233	56.3737636	7.698149155	2.944511624	0.000903001	0.037625937	fatty acid binding protein 1, liver
Apobec2	42.86262647	59.65982294	26.06543001	0.436900894	-1.194622035	0.000904311	0.037625937	apolipoprotein B mRNA editing enzyme, catalytic polypeptide 2
Adrb2	268.839657	389.4105508	148.2687631	0.380751787	-1.393077287	0.00094639	0.039159599	adrenergic receptor, beta 2
Spsb2	364.4340408	453.7461827	275.1218988	0.606334355	-0.721814528	0.000947618	0.039159599	splA/ryanodine receptor domain and SOCS box containing 2
Fscn1	157.4024642	196.6367856	118.1681428	0.60094627	-0.734692089	0.000956058	0.039325608	fascin actin-bundling protein 1
Kctd6	316.519234	249.8456037	383.1928644	1.53371866	0.617033864	0.000970503	0.039655718	potassium channel tetramerisation domain containing 6
Tmprss9	73.11013884	112.1257004	34.09457726	0.304074598	-1.717502795	0.000987645	0.040175986	transmembrane protease, serine 9
Pappa2	74.65435415	25.56463492	123.7440734	4.840439685	2.275138102	0.001005762	0.040731145	pappalysin 2
Adam22	355.531544	277.4104724	433.6526155	1.563216456	0.644517559	0.001020526	0.041075301	a disintegrin and metallopeptidase domain 22
Ugt1a2	595.1414112	31.52887668	1158.753946	36.75214812	5.199756671	0.001025901	0.041075301	UDP glucuronosyltransferase 1 family, polypeptide A2
Pdzk1	16105.58546	20090.08439	12121.08653	0.603336765	-0.728964597	0.001033373	0.041075301	PDZ domain containing 1
Mfge8	1405.351369	1110.55329	1700.149448	1.530903077	0.614382947	0.001037626	0.041075301	milk fat globule-EGF factor 8 protein
Cd55	187.3763037	233.5639886	141.1886188	0.604496522	-0.726194056	0.001039642	0.041075301	CD55 molecule, decay accelerating factor for complement
Loxl2	349.8986789	423.013313	276.7840447	0.65431521	-0.611942287	0.001043464	0.041075301	lysyl oxidase-like 2
Eif5a2	270.844591	211.8322436	329.8569384	1.557161142	0.638918249	0.001043561	0.041075301	eukaryotic translation initiation factor 5A2
Hmha1	182.6792816	138.4216072	226.936956	1.639461935	0.713222406	0.001050318	0.041252151	histocompatibility (minor) HA-1
Nox4	11479.10303	8615.992596	14342.21346	1.664603735	0.735178779	0.001081865	0.04239979	NADPH oxidase 4
2310042D19Rik	99.53450852	128.0186542	71.0503628	0.555000076	-0.849440126	0.001085672	0.042457711	NA
Aadat	4384.999085	5716.110298	3053.887872	0.534259787	-0.904386662	0.001106695	0.043100881	aminoadipate aminotransferase
Ralgps2	676.2003346	818.7174439	533.6832254	0.65185276	-0.617381969	0.001106849	0.043100881	Ral GEF with PH domain and SH3 binding motif 2
Serpinh1	1579.184608	2019.776195	1138.593022	0.563722369	-0.826943278	0.001137099	0.044058593	serine (or cysteine) peptidase inhibitor, clade H, member 1
Klhdc9	60.45512067	39.83812305	81.0721183	2.035038604	1.025056162	0.001137979	0.044058593	kelch domain containing 9
Fbln2	80.33158415	51.18845753	109.4747108	2.138660082	1.096707199	0.001169367	0.044958972	fibulin 2
Golm1	299.3412405	235.4617666	363.2207145	1.542588929	0.625353662	0.001193687	0.045489176	golgi membrane protein 1
Cntnap5a	45.7790496	78.47680714	13.08129205	0.166689912	-2.584761302	0.001194412	0.045489176	contactin associated protein-like 5A
Lgals1	646.4102297	788.0111007	504.8093588	0.640611989	-0.642477296	0.001195638	0.045489176	lectin, galactose binding, soluble 1
Gabra3	231.4726462	177.9672336	284.9780588	1.601295098	0.679239203	0.001198868	0.045517031	gamma-aminobutyric acid (GABA) A receptor, subunit alpha 3
Slc5a1	1709.140232	2074.194419	1344.086046	0.648003887	-0.625925628	0.001220076	0.046225924	solute carrier family 5 (sodium/glucose cotransporter), member 1
Acpp	248.409297	191.6552055	305.1633885	1.592252022	0.671068704	0.001225431	0.046332482	acid phosphatase, prostate
Ccdc15	157.2875706	118.716962	195.8581793	1.649791032	0.7222833	0.00123376	0.04648325	coiled-coil domain containing 15
Galnt14	1251.031445	1554.605957	947.4569323	0.6094515	-0.714416679	0.001262334	0.046981936	UDP-N-acetyl-alpha-D-galactosamine:polypeptide N-acetylgalactosaminyltransferase 14
Plxnc1	71.74739949	48.91712486	94.57767411	1.933426676	0.951160052	0.001262465	0.046981936	plexin C1
Cml5	522.6770706	747.6008248	297.7533164	0.398278475	-1.328150584	0.001266173	0.046981936	NA
Galns	1939.676456	2399.123375	1480.229537	0.616987668	-0.69668644	0.001267625	0.046981936	galactosamine (N-acetyl)-6-sulfate sulfatase
Steap3	100.6326928	127.7823954	73.4829903	0.57506349	-0.798206848	0.001268389	0.046981936	STEAP family member 3
Lypla1	4771.194999	5873.394645	3668.995352	0.624680542	-0.678809502	0.001277452	0.04703089	lysophospholipase 1
Adamtsl1	152.2520384	97.76434207	206.7397347	2.114674229	1.08043543	0.001298526	0.047622982	ADAMTS-like 1
Mki67	127.0794423	163.9675291	90.19135542	0.550056196	-0.862349076	0.001298761	0.047622982	antigen identified by monoclonal antibody Ki 67
Tusc5	52.9319606	33.82247609	72.04144511	2.129987317	1.09084484	0.001313699	0.047918642	tumor suppressor candidate 5
Abca5	929.5658767	713.5632747	1145.568479	1.605419616	0.682950431	0.001317342	0.047918642	ATP-binding cassette, sub-family A (ABC1), member 5
Sdc1	284.2154509	207.3187852	361.1121167	1.741820532	0.800595984	0.001342161	0.048434739	syndecan 1
Zfp97	107.969375	78.29600323	137.6427468	1.757979221	0.813918018	0.001348789	0.048577746	zinc finger protein 97
Car4	2722.662	3452.196675	1993.127326	0.577350456	-0.792480783	0.001359002	0.048849012	carbonic anhydrase 4
Tgfbi	502.0980416	399.6946845	604.5013986	1.5124079	0.596847291	0.001370248	0.049088033	transforming growth factor, beta induced
Cyp2j13	7826.408536	11393.41485	4259.40222	0.373847725	-1.419477342	0.001383121	0.049423533	cytochrome P450, family 2, subfamily j, polypeptide 13
Gcgr	329.3982834	398.3681849	260.4283819	0.653737903	-0.613215749	0.001390848	0.049602384	glucagon receptor
Siah2	406.699827	504.645141	308.7545129	0.611824999	-0.708809039	0.001400203	0.049679438	seven in absentia 2
Nap1l5	135.176347	72.91854338	197.4341507	2.707598664	1.43701391	0.001400678	0.049679438	nucleosome assembly protein 1-like 5

Supplementary Table 2

(Top hits on RNA Seq and their possible functions)

Gene Symbol	Gene Name	Gene Ontology; Biological Process	Gene Ontology; Molecular Function	InterPro	KEGG Pathways
Dbp	D site albumin promoter binding protein(Dbp)	GO:0001889~liver development, GO:0006351~transcription, DNA-templated,<br>GO:0006355~regulation of transcription, DNA-templated,<br>GO:0006357~regulation of transcription from RNA polymerase II promoter	GO:0000977~RNA polymerase II regulatory region sequence-specific DNA binding,	IPR004827:Basic-leucine zipper domain,	
Cndp2	CNDP dipeptidase 2 (metallopeptidase M20 family)(Cndp2)	GO:0006508~proteolysis, GO:0008152~metabolic process, GO:0043171~peptide catabolic process,	GO:0004180~carboxypeptidase activity, GO:0008233~peptidase activity, GO:0008237~metallopeptidase activity,	IPR001261:ArgE/DapE/ACY1/CPG2/YscS, conserved site	mmu00330:Arginine and proline metabolism, mmu00340:Histidine metabolism
Clca1	chloride channel accessory 1(Clca1)	GO:0006508~proteolysis,GO:0006810~transport,<br>GO:0006811~ion transport,<br>GO:0006816~calcium ion transport,<br>GO:0006821~chloride transport,<br>GO:0071456~cellular response to hypoxia,	GO:0005229~intracellular calcium activated chloride channel activity,<br>GO:0008237~metallopeptidase activity,<br>GO:0016787~hydrolase activity,<br>GO:0046872~metal ion binding,	IPR002035:von Willebrand factor, type A, IPR004727:Calcium-activated chloride channel protein,<br>IPR013642:Chloride channel calcium-activated,	mmu04924:Renin secretion,mmu04972:Pancreatic secretion,
Ypel2	yippee-like 2 (Drosophila)(Ypel2)		GO:0046872~metal ion binding,	IPR004910:Yippee-like protein,	
Arntl	aryl hydrocarbon receptor nuclear translocator-like(Arntl)	GO:0000060~protein import into nucleus, translocation,<br>GO:0006351~transcription, DNA-templated,<br>GO:0007283~spermatogenesis, GO:0007623~circadian rhythm, GO:0032007~negative regulation of TOR signaling	GO:0000976~transcription regulatory region sequence-specific DNA binding, GO:0000982~transcription factor activity, RNA polymerase II core promoter proximal region sequence-specific binding	IPR000014:PAS domain, IPR001067:Nuclear translocator,<br>IPR001610:PAC motif,<br>IPR011598:Myc-type, basic helix-loop-helix (bHLH) domain,<br>IPR013767:PAS fold,	mmu04710:Circadian rhythm, mmu04728:Dopaminergic synapse
Gjb2	gap junction protein, beta 2(Gjb2)	GO:0007154~cell communication, GO:0007267~cell-cell signaling, GO:0007605~sensory perception of sound, GO:0016264~gap junction assembly	GO:0005243~gap junction channel activity,<br>GO:0005515~protein binding,	IPR000500:Connexin,<br>IPR002268:Gap junction beta-2 protein (Cx26),<br>IPR013092:Connexin, N-terminal,<br>IPR019570:Gap junction protein, cysteine-rich domain,	
Nfil3	nuclear factor, interleukin 3, regulated(Nfil3)	GO:0000122~negative regulation of transcription from RNA polymerase II promoter,<br>GO:0006351~transcription, DNA-templated,<br>GO:0006355~regulation of transcription, DNA-templated	GO:0000977~RNA polymerase II regulatory region sequence-specific DNA binding,<br>GO:0000979~RNA polymerase II core promoter sequence-specific DNA binding,<br>GO:0001078~transcriptional repressor activity, RNA polymerase II core promoter proximal region sequence-specific binding	IPR004827:Basic-leucine zipper domain,<br>IPR010533:Vertebrate interleukin-3 regulated transcription factor,<br>IPR016743:Transcription factor, basic leucine zipper, E4BP4,	
Slc16a14	solute carrier family 16 (monocarboxylic acid transporters), member 14(Slc16a14)	GO:0006810~transport,<br>GO:0055085~transmembrane transport,	GO:0008028~monocarboxylic acid transmembrane transporter activity,<br>GO:0015293~symporter activity,	IPR011701:Major facilitator superfamily,<br>IPR020846:Major facilitator superfamily domain,	
Gm906	predicted gene 906(Gm906)			IPR008979:Galactose-binding domain-like,	

Note: 1) Gene annotation were performed using DAVID (https://david.ncifcrf.gov/), Huang DW, Sherman BT, Lempicki RA. Systematic and integrative analysis of large gene lists using DAVID Bioinformatics Resources. Nature Protoc. 2009;4(1):44-57.

2) Interpro: Classifiction of protein familes, https://www.ebi.ac.uk/interpro/

3) KEGG: Kyoto Encyclopedia of Genes and Genomes, https://www.genome.jp/kegg/
