# Supplementary material for: Chloride channel accessory 1 integrates chloride channel activity and mTORC1 in aging‐related kidney injury
Source: Aging Cell. 2021 Jun 12;20(7):e13407. doi: 10.1111/acel.13407 (PMC8282273; doi:10.1111/acel.13407)
Supplement: Supplementary file 2 — Supplementary Material [file ACEL-20-e13407-s002.docx]

**Supplemental Material Table of Contents**

**Supplementary Figures**

**S1. Aging is associated with increased kidney CLCA1 expression in marmosets.** Immunoblotting of kidney cortex of young and aged female marmosets showed increased expression of the 72 kDa fragment of CLCA1, probably the N-terminal fragment. A trend toward increase in the expression of the whole 130 kDa molecule was seen in the same animals.

**S2.** Rapamycin abolished the increase in phosphorylation of p70S6 kinase in hCLCA1 overexpressing cells indicating inhibition of mTORC1.

**Supplementary Table**

**Table 1.** List of mRNAs that were differentially regulated in renal cortex of aged mice.

**Table 2.** Top hits on RNA-Seq and their possible functions.
